# Mitochondrial Localization and Function of Adenosine Receptors

**DOI:** 10.7150/ijbs.101930

**Published:** 2025-02-10

**Authors:** Alejandro Sánchez-Melgar, Valentina Vultaggio-Poma, Simoneta Falzoni, Clara Fructuoso, José Luis Albasanz, Francesco Di Virgilio, Mairena Martín

**Affiliations:** 1 Department of Inorganic, Organic Chemistry and Biochemistry. Faculty of Medicine of Ciudad Real / Faculty of Chemical Sciences and Technologies. Institute of Biomedicine (IB-UCLM). IDISCAM. University of Castilla-La Mancha. Ciudad Real, Spain.; 2 Department of Medical Sciences, University of Ferrara, Ferrara, Italy.

**Keywords:** adenosine receptors, mitochondrial morphology, intracellular GPCR, ATP production

## Abstract

G-protein coupled receptors (GPCRs) are typically expressed on the cell surface where they mediate extracellular signals from hormones, neurotransmitters and growth factors, among others. However, growing evidence support the intracellular localization of GPCRs, including mitochondria. In the present work, we assessed the presence and functionality of adenosine receptors in mitochondria by combining techniques such as western blotting, radioligand binding, electron microscopy, enzymatic activities determination, oxygen consumption measurement and 3D morphological analysis of mitochondrial networks. Our results demonstrate the mitochondrial localization of adenosine A_1_ and A_2_ receptors in pure mitochondria fractions isolated from mouse brain and liver, human brain, HeLa and SH-SY5Y cells. Adenylyl cyclase activity assays revealed that these receptors are functional in the mitochondria. Moreover, exposure of isolated mitochondria to selective A_1_, A_2A_ and A_2B_ receptors agonists revealed these receptors as potential modulators of mitochondrial energy metabolism, since ATP production and coupling efficiency increased in the presence of BAY 60-6583 (A_2B_ agonist) whereas proton leak and acute response were higher with CGS 21680 (A_2A_ agonist). Also, proton leak, ATP production, acute response and acute respiration were increased in the presence of CPA (A_1_ agonist). Interestingly, different mitochondrial morphological changes were detected in HeLa cells exposed to these receptors' agonists.

## Introduction

G-protein coupled receptors (GPCRs) are typically expressed on the cell surface where they mediate extracellular signals from hormones, neurotransmitters and growth factors, among others. Many GPCRs, through the activation or inhibition of adenylyl cyclase (AC) or Phospholipase C (PLC), can fine-tune regulate the level of second messengers such as cyclic AMP (cAMP) and IP_3_/Ca^2+^, respectively, thus modulating multiple cellular signaling pathways and biological processes 1, 2. Adenosine receptors, belonging to the class A family of GPCR, are widely distributed throughout the body displaying many diverse biological functions, ranging from neuromodulation to immune system regulation. Four adenosine receptor subtypes are known: A_1_ and A_3_ are coupled to inhibitory G-proteins and promote adenylyl cyclase inhibition, whereas A_2A_ and A_2B_ are coupled to stimulatory G-proteins that, in turn, raise cAMP levels by activating adenylyl cyclase 3.

In the last few years, growing evidence supports the intracellular localization of GPCRs, including nucleus, endoplasmic reticulum, Golgi apparatus, lysosomes and mitochondria 4. The presence of GPCRs in intracellular organelles may trigger signaling events distinct from the canonical responses initiated at the cell surface 5. In line with this, a revision of current models of GPCR signaling to include the effect of their subcellular localization has been proposed 6. Pioneering studies have reported in several tissues the mitochondrial localization of GPCRs, such as Type-1 Cannabinoid receptor (CB1) 7, 8, Melatonin receptor 1 (MT1) 9, Angiotensin receptors 1 and 2 (AT1 and AT2) 10, the orphan GPR35 11 and purinoreceptors P2Y1, P2Y2 and P2Y12 12.

Mitochondria, the “powerhouse” of eukaryotic cells, supply cells with fuel energy in the form of ATP 13. Besides, these organelles contribute to cell physiology by regulating key biological functions, including apoptosis 14 and cell cycle progression 15. The mitochondrial network can be regulated through fusion and fission events based on the different cellular requirements. Thus, a high fusion rate which leads to interconnected mitochondria will facilitate ATP production, while mitochondrial fission will hamper ATP generation 16. Aging and several pathologies, including cancer and neurodegenerative disorders, present mitochondrial dysfunction, which therefore has become a recent hot focus of investigation. Likewise, it has been demonstrated that the mitochondrial AC/cAMP/PKA signaling cascade may serve as a metabolic sensor to modulate oxidative phosphorylation and thus ATP production as well as ROS generation 17, 18. In this line, additional studies suggested that intramitochondrial cAMP and its downstream effector PKA are associated with a higher activity of respiratory chain-complex I 17. This is of particular interest since AC/cAMP/PKA signaling cascade is involved in the transduction pathway of many GPCRs. Therefore, the present work aimed to assess the presence of adenosine receptors in mitochondria and to figure out their role in the modulation of mitochondrial functions.

## Materials and methods

### Reagents

All reagents used were of analytical grade. Details on specific materials and reagents can be found in the corresponding method section.

### Mouse strain and tissue extraction

C57BL/6 mice (3 months old) were fed with a standard rodent chow diet and water *ad libitum.* The animals were maintained at the animal facility at 22-24°C with a 12 h light cycle in a specific pathogen-free environment. Euthanasia was carried out by cervical dislocation to avoid mitochondrial damage. Animal experimentation was carried out under authorization from the Italian Ministry of Health (n. 744/2018-PR and n. 264/2021-PR). Brain and liver tissues were quickly collected upon euthanasia and maintained at 4°C in 50 mL tubes containing freshly prepared buffer (250 mM sucrose, 10 mM Tris/MOPS and 1 mM EGTA/Tris, pH 7.4).

### Cell culture

Human HeLa cervical carcinoma cells were obtained from the American Type Culture Collection (CCL-2). SH-SY5Y human neuroblastoma cells were obtained from American Type Culture Collection (CRL-2266). These cell lines were maintained in DMEM (Dulbecco's Modified Eagle's Medium), supplemented with 10% fetal bovine serum (Biowest, ref. S181B-500, Labclinics, Madrid, Spain) and 1% antibiotic-antimycotic (Gibco, NY, USA) and grown in a humidified atmosphere with 5% CO_2_ at 37°C.

### Human tissue

Human brain samples were obtained from the Institute of Neuropathology HUB-ICO-IDIBELL Biobank following the Spanish legal regulations (RD 1716/2011) and the approval of the local ethics committee of the Bellvitge University Hospital. The parietal cortex from brains of control patients was obtained and immediately prepared for morphological and biochemical studies. Special attention was paid to minimize limitations related to molecular studies of the *postmortem* brains including combined pathologies, metabolic syndrome, medication, long agonic stress, reduced *postmortem* delay and controlled conditions of temperature and tissue processing that could interfere with biochemical studies, as detailed elsewhere 19.

### Isolation of highly purified mitochondria

Fresh mouse forebrain and liver were washed to remove the remaining blood and minced before homogenization. Human brain samples were also minced before homogenization. For HeLa and SH-SY5Y cells, twenty confluent petri dishes were used. Samples were transferred onto 15 mL glass/Teflon potter for homogenization in an isolation buffer containing 225 mM sucrose, 75 mM mannitol, 1 mM EGTA and 5 mM HEPES at pH 7.4. The number of strokes was different depending on the tissue to preserve mitochondrial integrity in each tissue (20 slow up and down strokes for mouse and human brain, 10 strokes for mouse liver and 40 strokes for HeLa and SH-SY5Y cells). Moreover, 0.1 µg/ml of digitonin was added to the human brain and HeLa and SH-SY5Y cells samples to facilitate plasma membrane disruption during the homogenization step without affecting mitochondrial integrity. Homogenate (H) was centrifuged at 600 x g for 5 min at 4ºC. The supernatant was collected and further centrifuged twice at 7000 x g for 10 min at 4ºC to obtain crude mitochondria (CM). The crude mitochondria fraction was resuspended in Percoll and transferred onto a clear-ultracentrifuge tube. The procedure for isolating pure mitochondria (PM) was carried out by using a Percoll-gradient ultracentrifugation as described elsewhere 20. All the procedure was performed at 4ºC to minimize activation of damaging proteases and phospholipases. The band located between 40% and 24% of Percoll was considered as pure mitochondria fraction (PM). All samples were stored at -80ºC until experiments were performed.

### Pure mitochondria fractionation

**Scheme 1:**
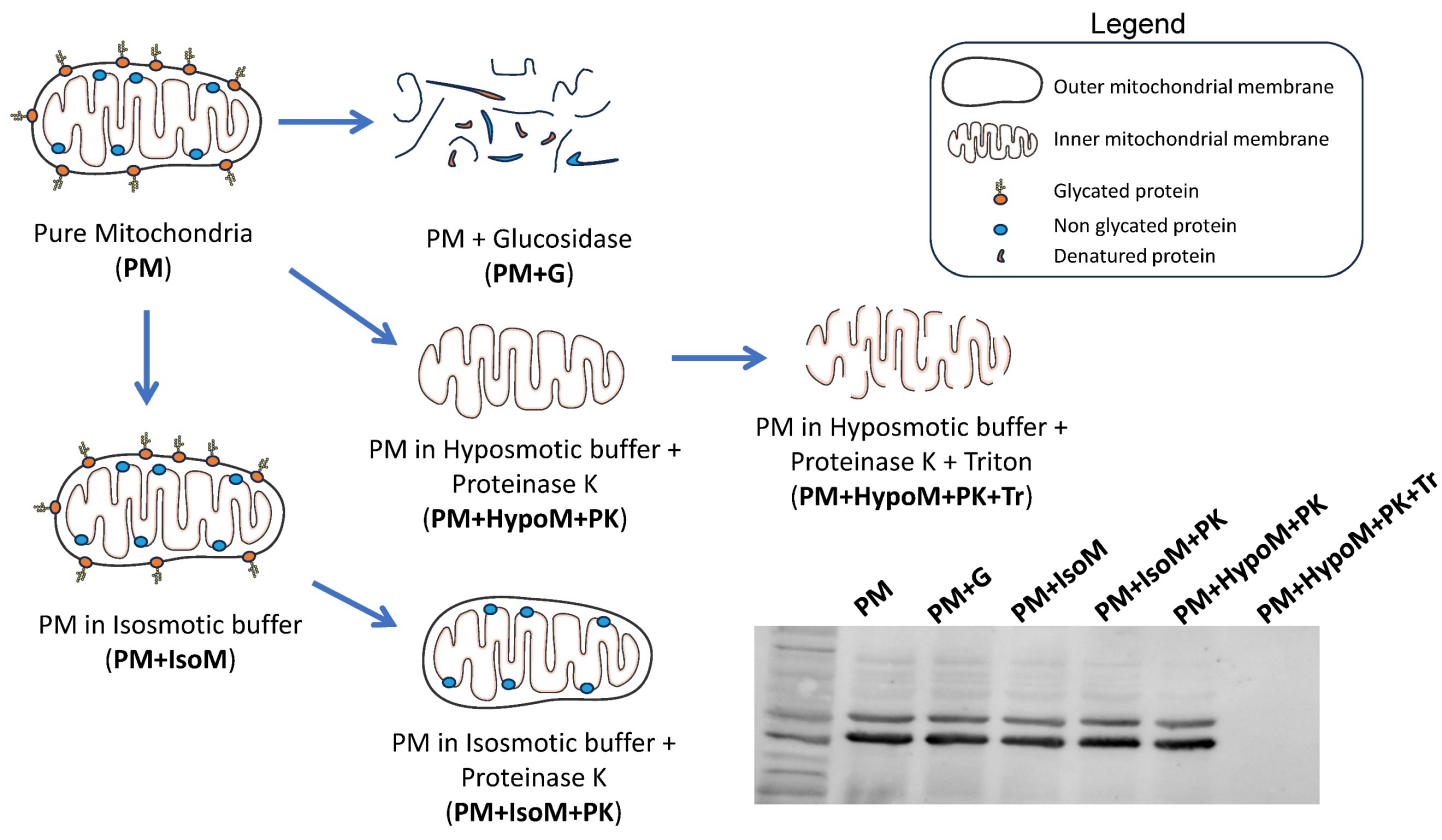
** Fractionation of pure mitochondria preparations.** Freshly isolated pure mitochondria (PM) were resuspended in different buffers (IsoM, isosmotic; HypoM, hypoosmotic) with or without proteinase K (PK) to analyze the presence of adenosine receptors in the mitochondrial membranes, inner and outer mitochondrial membrane. G, glucosidase. Tr, Triton X-100.

Pure mitochondria isolated from HeLa and SH-SY5Y cells were re-suspended in different buffers (Scheme 1): isosmotic buffer (225 mM Sucrose, 75 mM D-mannitol, 1 mM EGTA, 5 mM HEPES, pH 7.4) with or without proteinase K (PK, 100 µg/ml), hyposmotic buffer (10 mM Tris-MOPS, 0.5 mM EGTA, pH 7.4) plus PK (100 µg/ml) and hyposmotic buffer plus PK (100 µg/ml) and 0.1% v/v triton X-100. Samples were incubated on ice for 30 min and after this, proteinase K was blocked by adding 2 mM PMSF and incubated for an additional 5 minutes on ice. Samples were centrifuged at 14000g for 15 min at 4°C and the obtained pellet was used for western blot analysis. Pure mitochondria subfractions were resuspended in Laemmli loading buffer containing 10% β-mercaptoethanol, heated at 95ºC for 5 minutes and separated by SDS-PAGE 39. Additionally, some pure mitochondria fractions (PM+G) were incubated with an Endoglycosidase H from *Streptomyces plicatus* (EMD Millipore, ref. 324717) for 3 h at 37ºC with gentle shaking to remove glycosylation from proteins.

### Western blotting analysis

Fifteen micrograms of protein from each sample were mixed with loading buffer (0.125 M Tris-HCl, pH 6.8, 10% β-mercaptoethanol, 20% glycerol, 4% SDS, and 0.002% bromophenol blue) and heated for 5 min at 50°C. Sample proteins were separated on a 10% SDS-PAGE gel using a mini-protean system (Bio-Rad, Madrid, Spain) and transferred to nitrocellulose membranes in an iBlot Dry Blotting System (Invitrogen, Madrid, Spain). After washing with PBS Tween 20 and blocking with the same buffer containing 5% skimmed milk for 1 h at room temperature, membranes were incubated overnight at 4°C with the following primary antibodies: 1:1,000 anti-A_2B_ from Merck-Millipore (ab1589p); 1:1,000 anti-A_2A_ from Invitrogen (ab2257858); 1:2,000 anti-A_1_ from Abcam (ab124780); 1:2000 anti-Na^+^/K^+^ ATPase from Abcam (ab7671); 1:2,000 anti-VDAC from Antibodies Online (ABIN1305043); 1:1,000 anti-TOM20 from Abcam (ab186734); 1:2,000 anti-β-tubulin from Merck-Millipore (05-661). After three washes of 10 min, membranes were incubated with the corresponding GAR-PO or GAM-PO secondary antibodies (Bio-Rad, Madrid, Spain) at a dilution of 1:4,000 for 1h at room temperature. The ECL chemiluminescence detection kit (Amersham, Madrid, Spain) was used to visualize protein bands that were quantified using a G:Box system and GeneTools software (Syngene, Bristol, UK).

### Electron microscopy and immunogold labelling of mitochondria

Mouse brain was minced in small pieces and transferred into a glass/Teflon potter for manual homogenization in freshly prepared buffer (250 mM sucrose, 10 mM Tris/MOPS, and 1 mM EGTA/Tris, pH 7.4) on ice. Only 5-6 slow up-and-down strokes were enough to homogenize brain tissue to preserve mitochondrial integrity and morphology. Brain homogenate was then centrifuged at 600 x g for 5 min at 4ºC to remove unbroken cells/tissue and nuclei. The supernatant was collected and centrifuged twice at 7,000 x g for 10 min at 4ºC to obtain a mitochondria crude fraction. The obtained pellet was then fixed with 2% paraformaldehyde for 1 h at room temperature. The fixed pellet was washed with PBS and centrifuged twice at 7,000 x g for 10 min to remove the remaining fixative and then incubated with PBS with 0.05% Triton X-100 for 20 min at room temperature to permeabilize the mitochondria. After permeabilization, samples were incubated for 20 min with 2% BSA in PBS to block non-specific sites. Primary antibodies anti-A_2B_ from Merck-Millipore (ab1589p), anti-A_2A_ from Santa-Cruz Biotechnology (sc-32261), and anti-TOM20 from Sigma-Aldrich (HPA011562) were added to the sample at a dilution of 1:20 with 0.2% BSA and was incubated overnight at 4ºC. Bound primary antibodies were detected with protein A coated with 20-nm gold (Abcam, ref. ab270601) at the final dilution of 1:20 in 0.2% BSA-containing PBS 38984997

### Radioligand binding assay

Binding assays in highly purified mitochondria fraction obtained from the liver were performed as described previously 21. Briefly, samples were incubated with 5 U/mg adenosine deaminase in 50 mM Tris-HCl, 2 mM MgCl_2_, pH 7.4, for 30 min at 25°C to remove endogenous adenosine. Mitochondria fraction (50 μg protein) was then incubated at a concentration of saturation of 20 nM [^3^H]DPCPX or 20 nM and 50 nM [^3^H]ZM 241385, a selective antagonist of A_1_ and A_2A_ receptors, respectively, for 2 h at 25°C. Non-specific binding was obtained by using 1 mM CPA and 0.5 mM ZM 241385 or 9 mM Theophylline as displacing ligands for A_1_ and A_2A_ receptors, respectively. Binding assays were stopped by rapid filtration through Whatman GF/ B filters, previously pre-incubated with 0.3% polyethylenimine using a FilterMate Harvester (PerkinElmer). Radioactivity was measured in a Microbeta Trilux (PerkinElmer) liquid scintillation counter.

### Adenylyl cyclase activity

Adenylyl cyclase activity was determined as previously described 21 with minor changes. Thirty micrograms of protein from each sample were added into a tube in a final volume of 0.25 mL of 50 mM Tris-HCl, pH 7.4, containing 5 mM MgCl_2_, 1 mM 1,4-dithiothreitol, 1 mg/mL bovine serum albumin, 1 mg/mL creatine kinase, 10 mM creatine phosphate and 0.1 mM BAY 60-7550 (a specific phosphodiesterase inhibitor). Highly purified mitochondria fractions from mouse liver, previously treated with adenosine deaminase (5 U/mg protein) for 30 min at 37°C to remove endogenous adenosine, were incubated for 10 min at 37°C in the absence or the presence of 50 μM forskolin, 10 μM CPA, 10 μM CGS 21680 and 10 μM of BAY 60-6583. The reaction was initiated by adding 2 mM ATP at 37°C for 30 min. The enzymatic activity of adenylyl cyclase was stopped by boiling the samples. Tubes were centrifuged for 4 min at 12,000 x g. 50 µl of supernatant was used to determine cAMP accumulation. Samples were incubated with 0.25 pmol of [^3^H]cAMP and 6.25 μg protein kinase A in a final volume of 200 μL of assay buffer (50 mM Tris-HCl, pH 7.4, 4 mM EDTA) for 4 h on ice. The standard curve of cAMP was prepared in the same buffer ranging from 0 to 32 pmol and determined in parallel. Radioactivity was measured in a Microbeta Trilux (PerkinElmer) liquid scintillation counter.

### Determination of CD73 activity

Highly purified mitochondria fractions from liver and HeLa cells were used to measure the activity of 5'-ectonucleotidase by following the protocol previously described 21 with minor changes. Mitochondria fractions (30 µg of protein) were pre-incubated in 180 µl of the reaction buffer containing 50 mM Tris, 5 mM MgCl_2_, pH 9 for 10 min at 37°C. The reaction was then initiated by adding 20 µl of AMP at a final concentration of 0.5 mM and stopped 20 min later by adding 200 µl of 10% trichloroacetic acid. Samples were cooled down on ice for 10 min and then centrifuged at 12,000 x g for 4 min at 4°C. The resulting supernatants were collected to quantify the inorganic phosphate released. 0.5 mM AMP alone was also assayed in parallel to measure the non-enzymatic AMP degradation and subtracted from sample values. Furthermore, 0.1 mM levamisole, an inhibitor of the alkaline phosphatase, was used to ensure that the inorganic phosphate released was specific from 5'-ectonucleotidase activity.

### Measurement of mitochondrial respiration by Seahorse analysis

**Scheme 2:**
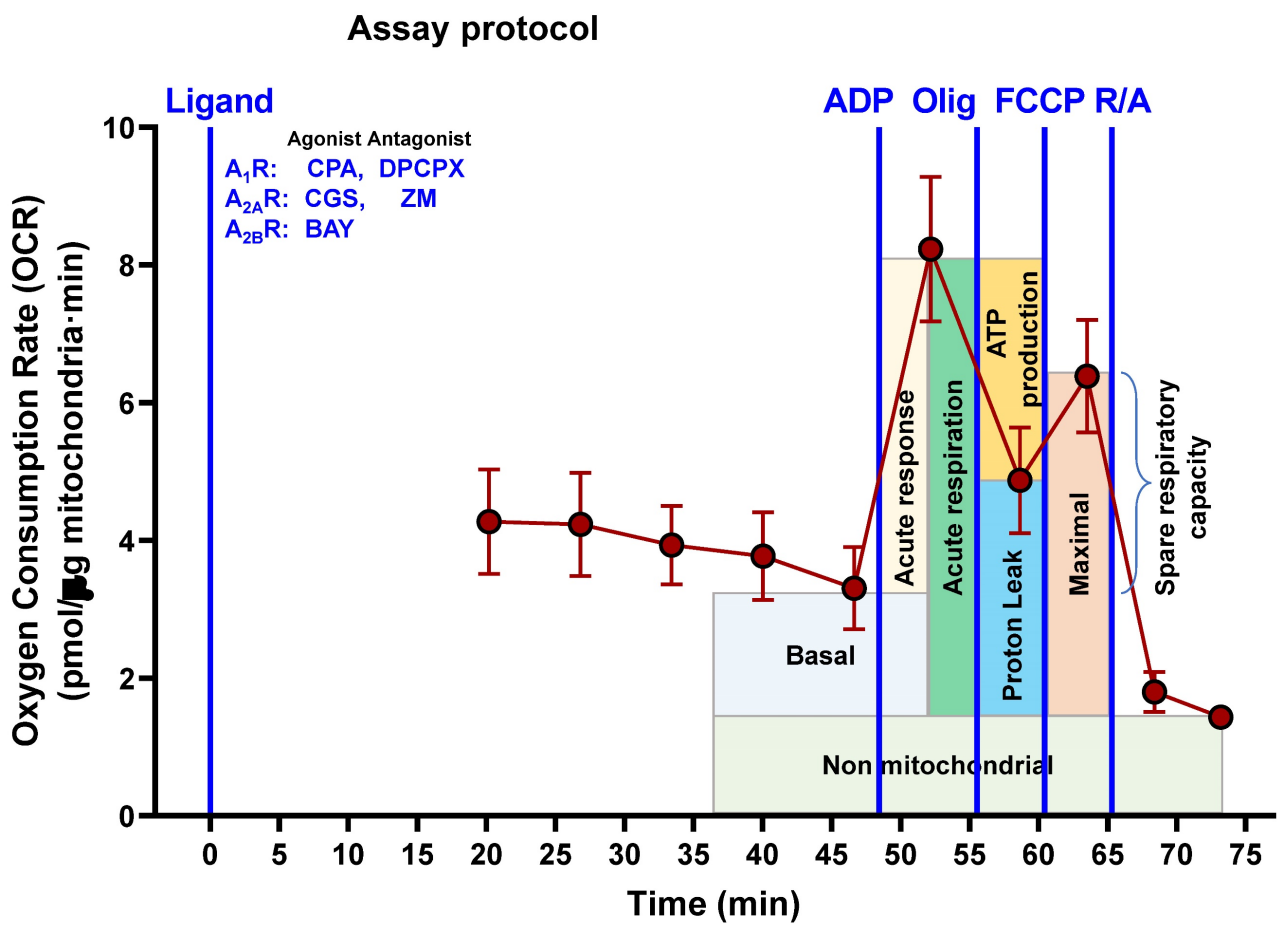
** Assay protocol followed to measure oxygen consumption by isolated mitochondria from HeLa cells.** Freshly isolated mitochondria were exposed to different adenosine receptor agonists (CPA, CGS, BAY) or antagonists (DPCPX, ZM). In the presence of these ligands, adenosine diphosphate (ADP), oligomycin (Olig), carbonylcyanide-p-trifluoromethoxyphenylhydrazone (FCCP) and rotenone/antimycin A (R/A) were sequentially injected in the reaction well at the indicated time and the corresponding oxygen consumption measured.

To avoid the possible effect of adenosine receptors located on the plasma membrane on mitochondrial functionality, isolated mitochondria from HeLa cells were used to assess the mitochondrial respiration in a SeaHorse XFp analyzer and its Seahorse Wave Pro Software (Agilent Technologies, Santa Clara, CA, USA). Assays were carried out by following the protocol described elsewhere 22 and based on the Cell Mito Stress Test assay kit for the SeaHorse XFp analyzer. Ten confluent petri dishes were needed to obtain the mitochondria crude fraction. Cells were detached and transferred into a glass/Teflon potter and homogenized with 40 strokes in MIB (Mitochondrial Isolation Buffer: 210 mM D-mannitol 70 mM sucrose, 5 mM HEPES, 1 mM EGTA, 0.5% free-lipid BSA, pH 7.2) containing 0.1 µg/ml of digitonin to disrupt plasma membrane without affecting mitochondrial integrity. Homogenate was centrifuged at 800 x g for 10 min at 4ºC. The supernatant was collected and further centrifuged twice at 8,000 x g for 10 min to obtain a crude mitochondria fraction that was resuspended in MAS (Mitochondrial Assay Solution: 220 mM D-mannitol, 70 mM sucrose, 10 mM KH_2_PO_4_, 5 mM MgCl_2_, 2 mM HEPES, 1 mM EGTA, 0.2% free-lipid BSA, pH 7,2) with 20 mM glutamate and 10 mM malate as mitochondrial substrates. Crude mitochondria (10 µg of protein) were added to each well of the 8-well XFp cell culture plate and then centrifuged at 1,200 x g for 30 min at 4ºC to promote adherence of mitochondria. Freshly prepared ten-fold concentrated compounds (40 mM ADP, 100 µM oligomycin, 100 µM FCCP and 50 µM of rotenone/Actinomycin A) were added to the ports of the cartridges, whereas selective ligands for adenosine receptors (final concentration of 10 µM CPA, 10 µM CGS 21680 and 10 µM BAY 60-6583, 10 µM DPCPX and 10 µM ZM 241385) were added to the corresponding well. The temporal assay protocol of injections (Scheme 2) allows the calculation of several parameters related to mitochondrial respiration (nonmitochondrial respiration, basal respiration, maximal respiration, ATP production, proton leak, acute response, and acute respiration). Two wells for each condition were run in parallel and data analysis of ΔOCR (Delta Oxygen Consumption Rate) was calculated as previously described 22.

### Analysis of mitochondrial morphology

**Scheme 3:**
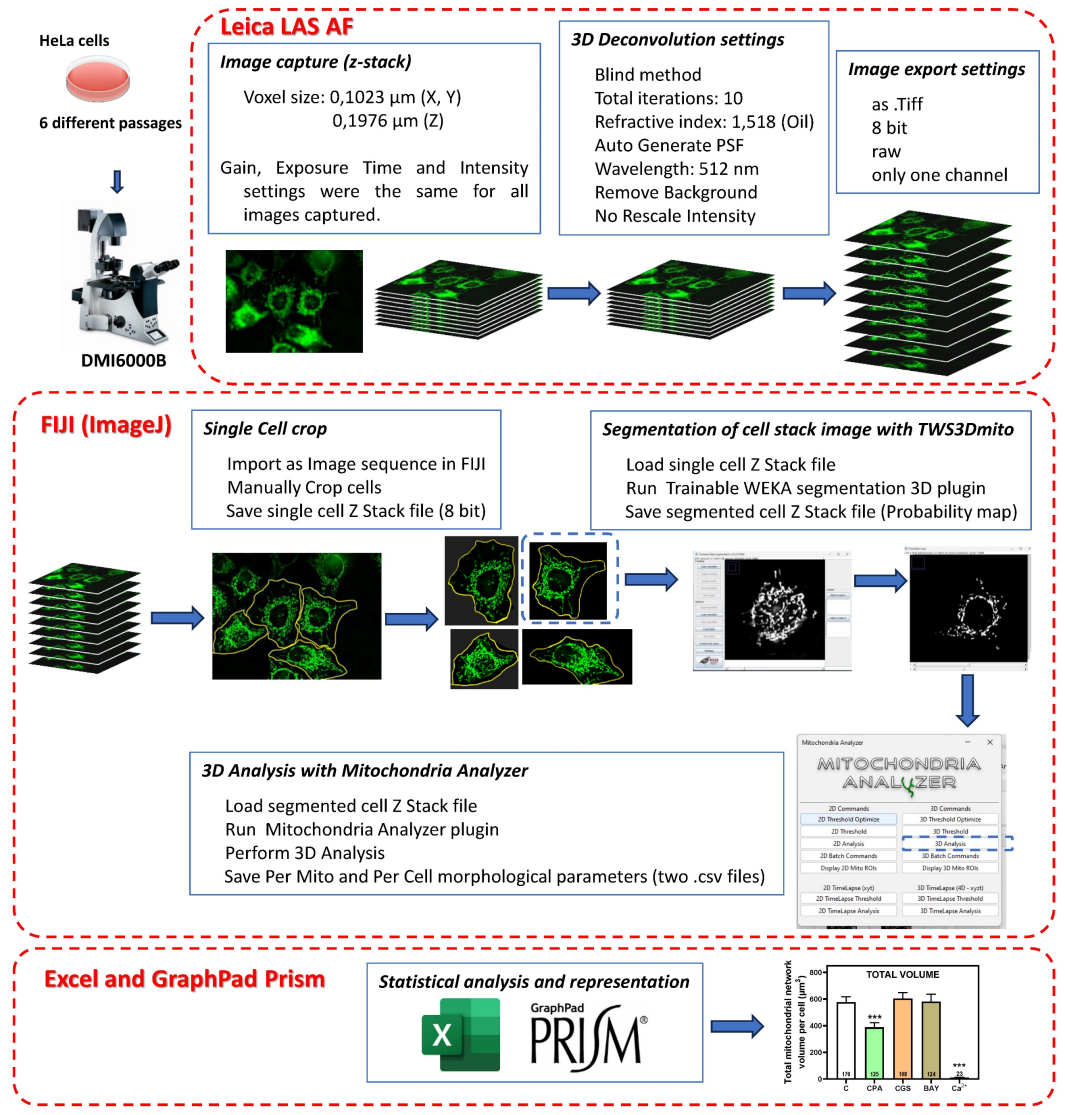
** Pipeline followed to measure mitochondrial 3D morphology in HeLa cells.** Cultured HeLa cells were exposed to Mitotracker Green FM to stain mitochondria and z-stack images were captured in a Leica DMI6000B microscope with a 63x immersion oil objective and LAS AF software. Resolution (0.1023 µm/pixel for X- and Y-axis; 0.1976 µm/pixel for Z-axis) allowed a voxel size of 0,00206 µm^3^. All acquisition parameters (gain, exposure time and intensity settings) were maintained constant for all assays performed. Images were deconvolved with Leica LAS AF software with parameters indicated in the figure and exported to be analyzed with FIJI (ImageJ) software (https://imagej.net/software/fiji/downloads) and the indicated plugins.

MitoTracker Green FM probe (Invitrogen, Waltham, Massachusetts, USA), which is independent of mitochondrial membrane potential, was used for labelling mitochondria. HeLa cells were seeded in 35 mm glass bottom dishes (Ibidi, Gräfelfing, Germany). After 24 h of exposure to different compounds (final concentration of 10 µM CPA, 10 µM CGS 21680, 10 µM BAY 60-6583, 10 µM Forskolin, and 10 µM Ca^2+^), the culture medium was removed from control or treated cells and replaced with a saline solution containing MitoTracker Green FM probe at a final concentration of 200 nM. Cells were then incubated at 37ºC for 20 minutes and visualized with a fluorescence microscope Leica DMI6000B (Leica Microsystems, Wetzlar, Germany) using a 63x immersion oil objective. Images were captured within a 30-35 min time frame after probe incubation to reduce the mitotoxicity induced by the fluorescent probe. Computer processing and image analysis of cell cultures were carried out as detailed in Scheme 3. The experiment was repeated with 6 independent cell cultures (different passage numbers and dates), each of them with the corresponding control and treated cells. Therefore, a total of 556 interphase cells were analyzed for mitochondria identification and 3D morphological characterization. Quantitative evaluation of mitochondria morphology requires a reliable segmentation of mitochondrial networks in microscopy images. This crucial step was performed with Trainable Weka Segmentation 3D, a machine learning-based 3D segmentation of mitochondria 23 that can be used as an ImageJ plugin (TWS3Dmito and resources are available on GitHub at github.com/MitoHultgren/TWS3Dmito). After the automatic 3D segmentation of complete mitochondrial networks, we quantitatively evaluated the resulting images using the 3D Analysis submenu of Mitochondrial Analyzer, a built-in ImageJ plugin, to detect and quantify 3D objects (mitochondria), which measures various shape descriptors of the mitochondrial network 24. After 3D object recognition, objects with a volume smaller than 0.1 μm^3^ were excluded to reduce artifacts 25.

### Statistical analysis

Data are the means ± SEM. Statistical analysis included Student's *t*-test. Differences between mean values were considered statistically significant at p < 0.05. GraphPad Prism 8.4 program was used for statistical and data analysis (GraphPad Software, San Diego, CA, USA).

## Results

### Adenosine receptors localize on mitochondria from several tissues

**Figure 1:**
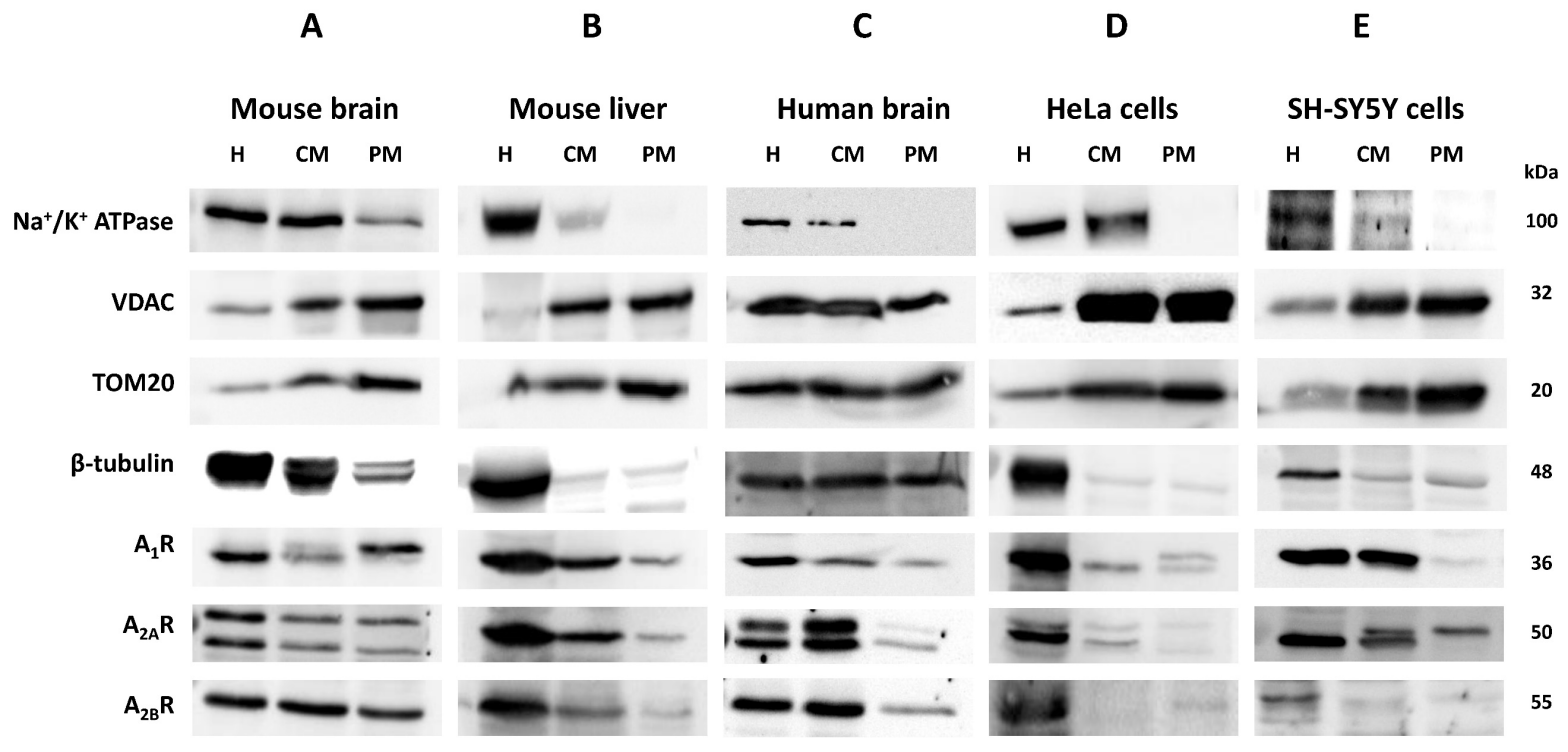

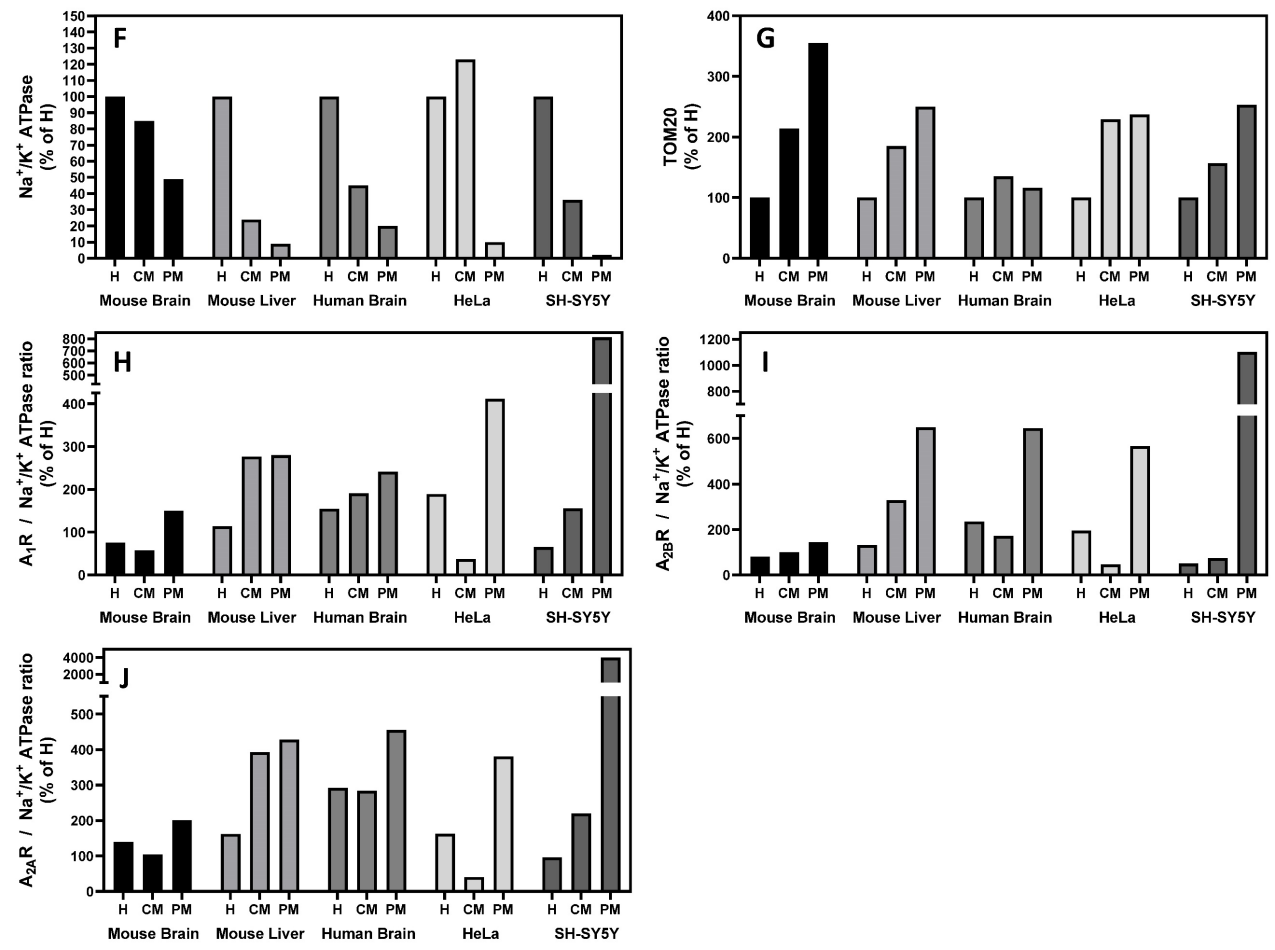
** Mitochondrial localization of adenosine receptors in several tissues.** Tissue homogenate (H), crude mitochondria fraction (CM), and highly purified mitochondria fraction (PM) were analyzed by immunoblotting of specific markers of plasma membrane (Na^+^/K^+^ ATPase), mitochondria (VDAC and TOM20) and cytosol (β-tubulin) from mouse brain (A) and liver (B), and human brain (C), HeLa cells (D) and SH-SY5Y cells (E) to verify the purity of the mitochondria fraction. Immunodetection of adenosine A_1_, A_2A_, and A_2B_ receptors was performed in the same tissues and fractions, and their presence was relativized to Na^+^/K^+^ ATPase levels (F-J). Blot images are representative of three to four independent experiments.

Homogenates (H), crude mitochondria (CM), and pure mitochondria fractions (PM) were isolated from different sources (i.e. mouse brain and liver, human brain, and human HeLa cells), and the purity of such fractions was verified by incubating with primary antibodies that target specific markers from different cellular compartments (i.e. Na^+^/K^+^ ATPase for plasma membrane, VDAC and TOM20 for mitochondria and β-tubulin for cytosol). Western blots confirmed the enrichment of the CM and PM fractions in mitochondria since an increased presence of TOM20 and VDAC, and a decreased Na^+^/K^+^ ATPase signal, were observed (Fig. 1F, G). Moreover, the absence of Na^+^/K^+^ ATPase signal revealed that PM fraction was highly enriched in mitochondria from mouse liver (Fig. 1B), human brain (Fig. 1C) and HeLa cells (Fig. 1D) and SH-SY5Y cells (Fig. 1E) with marginal contamination of cytosol and plasma membrane in mouse brain (Fig. 1A). A clear immunoreaction of A_1_, A_2A_ and A_2B_ receptors was found in CM and PM fractions from tissues analyzed (Fig. 1A-E), which suggests a mitochondrial localization of these receptors.

**Figure 2:**
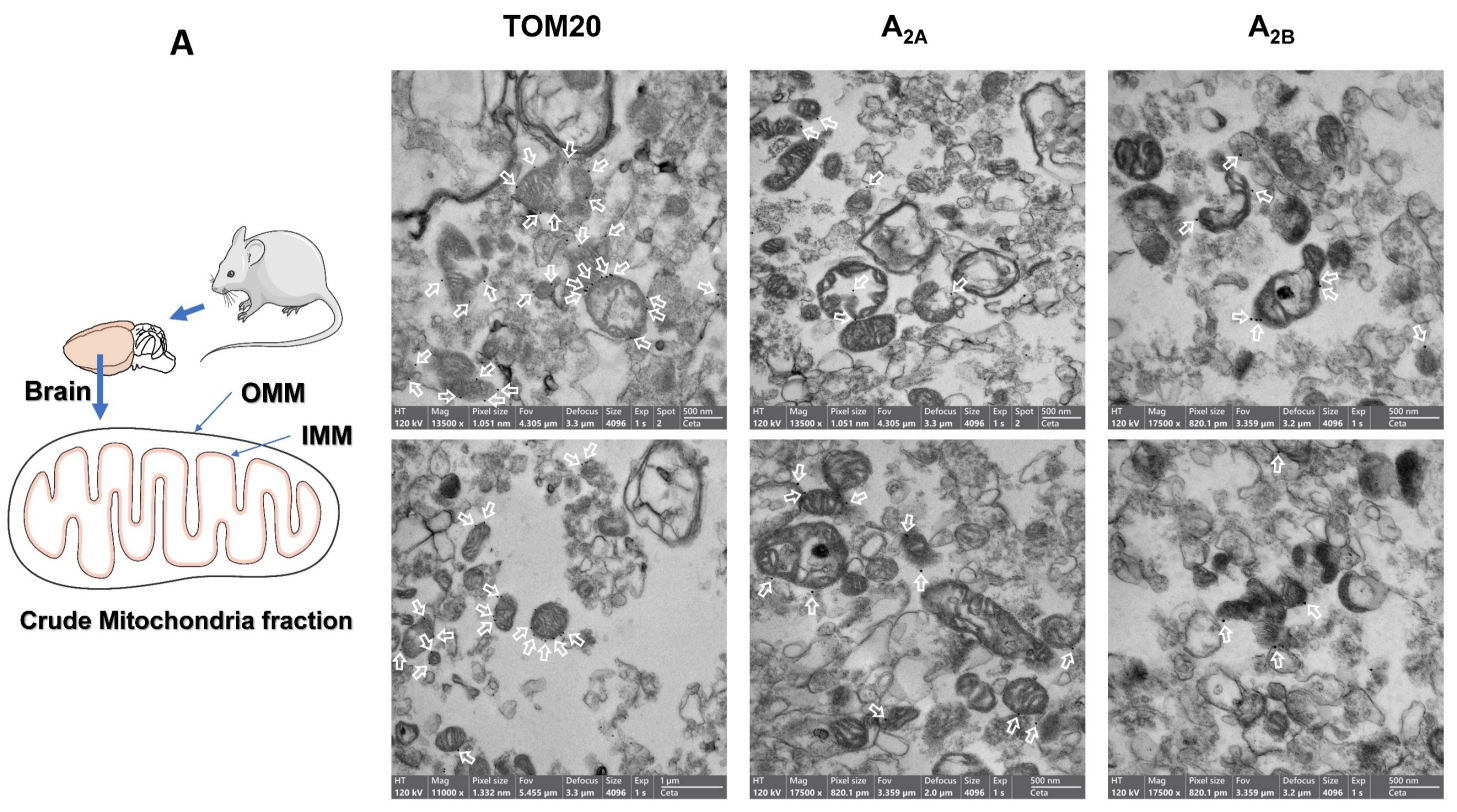

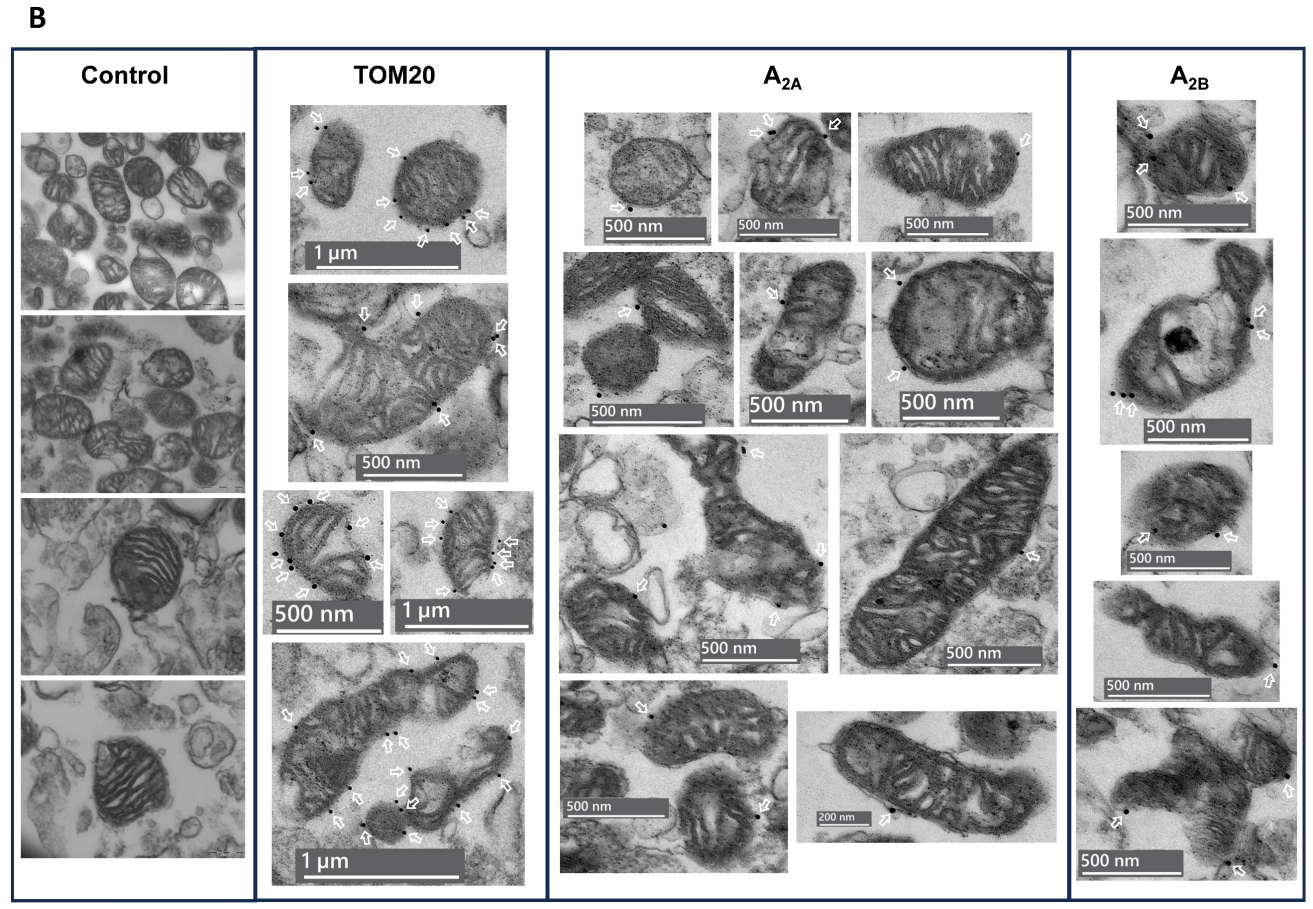
** Adenosine receptors are located on the outer mitochondrial membrane.** Electron microscopy (EM) imaging of mouse brain crude mitochondria fraction revealed the presence of adenosine receptors on the outer mitochondrial membrane. Representative general (A) or individual (B) mitochondria EM images with the corresponding scale bar size showing (white arrows) the immunogold staining of Tom20, as a positive control of mitochondria, and adenosine A_2A_ receptors (A_2A_) or A_2B_ receptors (A_2B_). These pictures are representative of four to five independent experiments.

The absence of Na^+^/K^+^ ATPase (a plasma membrane marker) in the PM fraction from mouse liver, human brain and HeLa and SH-SY5Y cells reinforces that adenosine receptors are present in the mitochondria. Moreover, an increased ratio adenosine receptor / Na^+^/K^+^ ATPase signal is obtained in the PM fraction confirming this presence (Fig. 1H-I). However, in the mouse brain, residual Na^+^/K^+^ ATPase contamination raises the possibility that the detected adenosine receptor signal in this tissue may be due to this membrane contamination and not its presence in mitochondria. Therefore, we tried to detect their mitochondrial presence by electron microscopy after immunogold labelling, the “gold standard” method for intracellular protein detection. Only adenosine A_2A_ and A_2B_ receptors were tested, and electron microscopy images corroborated their localization in mouse brain mitochondria (Fig. 2A). Anti-TOM20 was used as a positive control of mitochondria labelling. Since the mitochondria fraction used for immunogold assays was permeabilized with Triton X-100 to ensure free access of antibodies to the mitochondrial inner membrane, the immunolabelling observed in electron microscopy images strongly suggest a main localization of these receptors in the mitochondrial outer membrane (Fig. 2B).

**Figure 3:**
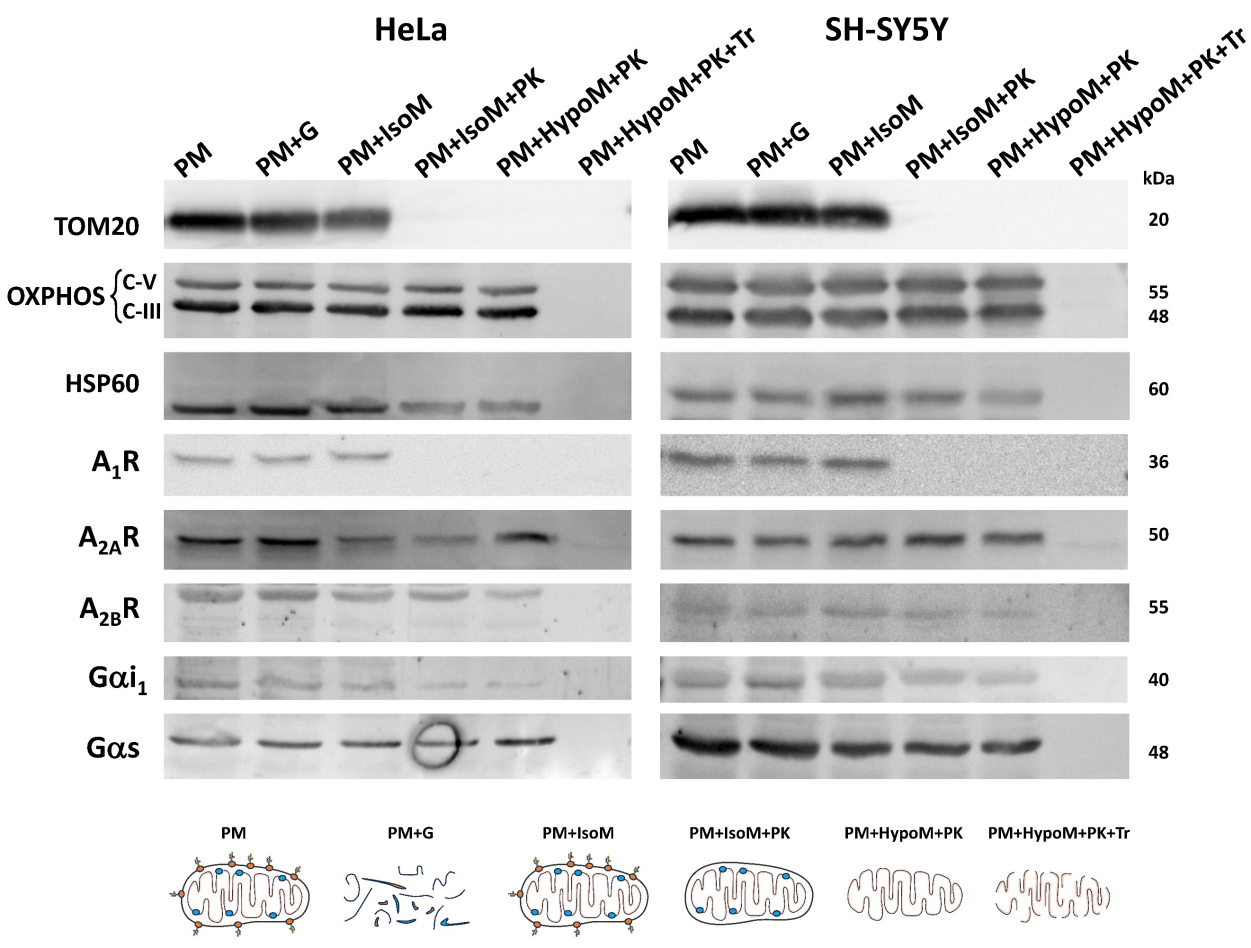
** Localization of adenosine receptors in mitochondrial membranes**. Western Blot analysis of pure mitochondria (PM) from HeLa and SH-SY5Y cells exposed to proteinase K (PK, 100 μg/mL) in iso- (IsoM) or hypo- (HypoM) osmotic buffer and stained with antibodies raised against the adenosine A_1_, A_2A_ and A_2B_ receptors and the G protein alpha s and i1 subunits. PM+G, denatured PM incubated with glucosidase; PM+IsoM, PM incubated in isosmotic medium; PM+IsoM+PK, PM incubated in isosmotic medium-plus PK; PM+HypoM+PK, PM incubated in hyposmotic medium plus PK. PM+HypoM+PK+Tr, PM incubated in hyposmotic medium plus Triton-X100 and PK. HSP60, OXPHOS, and TOM20 were used as markers of the mitochondrial matrix, the inner and the outer mitochondrial membrane, respectively.

The localization of adenosine receptors in the outer or the inner mitochondrial membrane was further explored by subfractionation and western blotting assays (Scheme 1). This mitochondrial subfractionation was performed in pure mitochondria fraction derived from HeLa and SH-SY5Y cells. As Figure 3 shows, the presence of A_2A_ and A_2B_ receptors was observed in both the outer mitochondrial membrane (OMM) and the inner mitochondrial membrane (IMM). However, the A_1_ receptor appears to be localized exclusively in the OMM. Removal of TOM20 (OMM marker) after proteinase K (PK) treatment confirmed the degradation of proteins from the OMM and exposed to PK action. OXPHOS complexes and HSP60 were used as IMM and mitochondrial matrix markers, respectively. Additionally, alpha i1 and alpha s G protein subunits were observed in both OMM and IMM.

**Figure 4:**
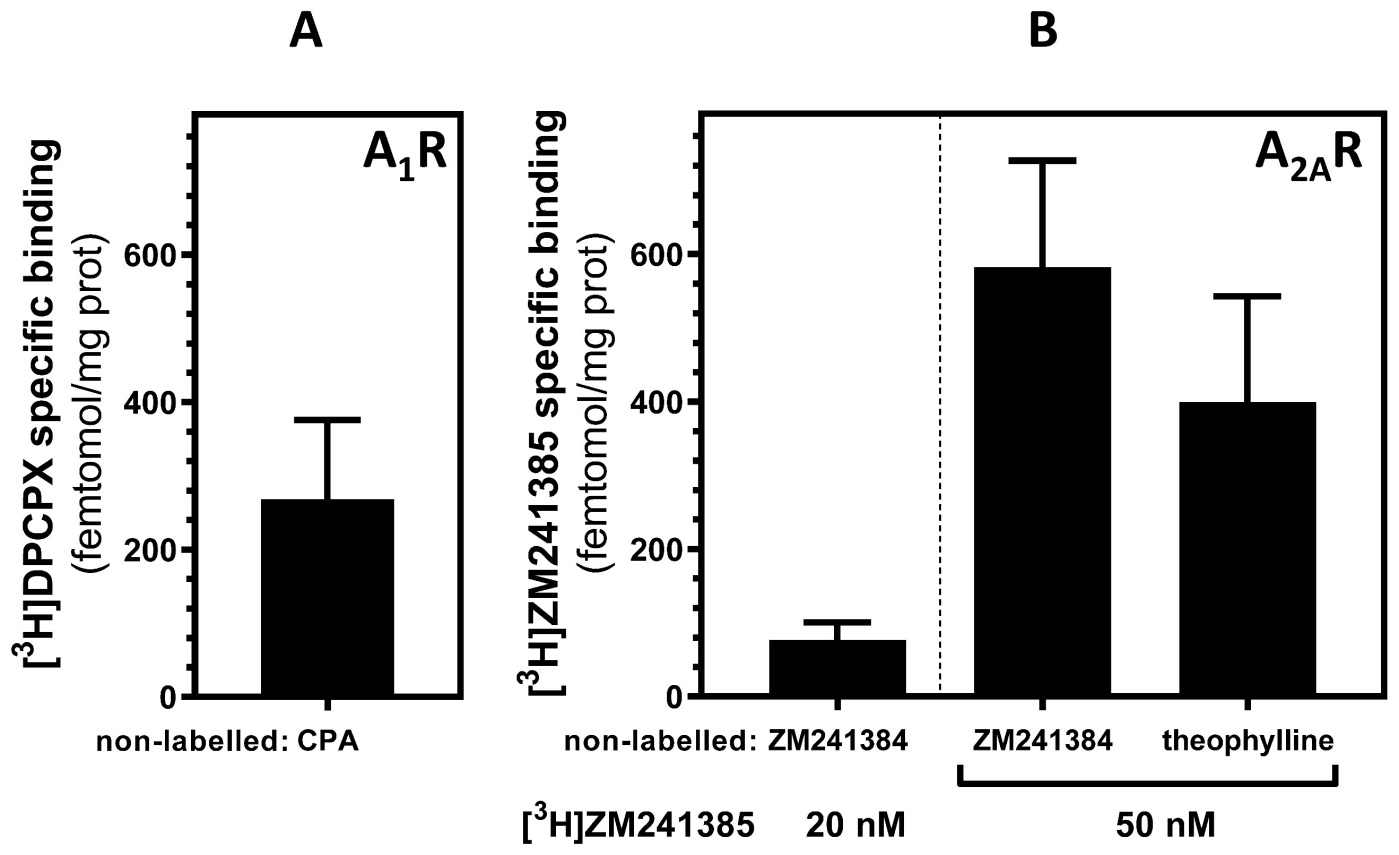
** Radioligand binding assay of adenosine A_1_ and A_2A_ receptors in mouse liver.** Highly purified mitochondria fraction was used for adenosine receptors quantification in mouse liver. Specific binding was determined at saturation concentrations of 20 nM [^3^H]DPCPX (A) and 20 or 50 nM [^3^H]ZM 241385 (B). 1 mM CPA and 500 µM ZM 241385 and 3 mM theophylline were used as displacing non-labelled ligands for A_1_ and A_2A_ receptors, respectively, to determine the non-specific binding. Data are the mean ± SEM of three independent assays.

Furthermore, to quantify adenosine receptors by using a non-antibody-based method, radioligand binding assay, a very sensitive technique, was carried out in PM fraction from mouse liver as the yield of PM fraction isolation was higher than in other tissues used. Results from the radioligand binding assay showed that both adenosine A_1_ and A_2A_ receptors (Fig. 4A and B, respectively) are in mouse liver mitochondria at a magnitude order of fmol /mg of protein. Interestingly, when PM fraction was treated with proteinase K, a reduction of 56% and 45% of specific A_1_ and A_2A_ receptor binding, respectively, was observed (data not shown) confirming again the presence of these receptors in the OMM.

### Adenosine receptors are coupled to their canonical G-protein and modulate cAMP levels in mitochondria

**Figure 5:**
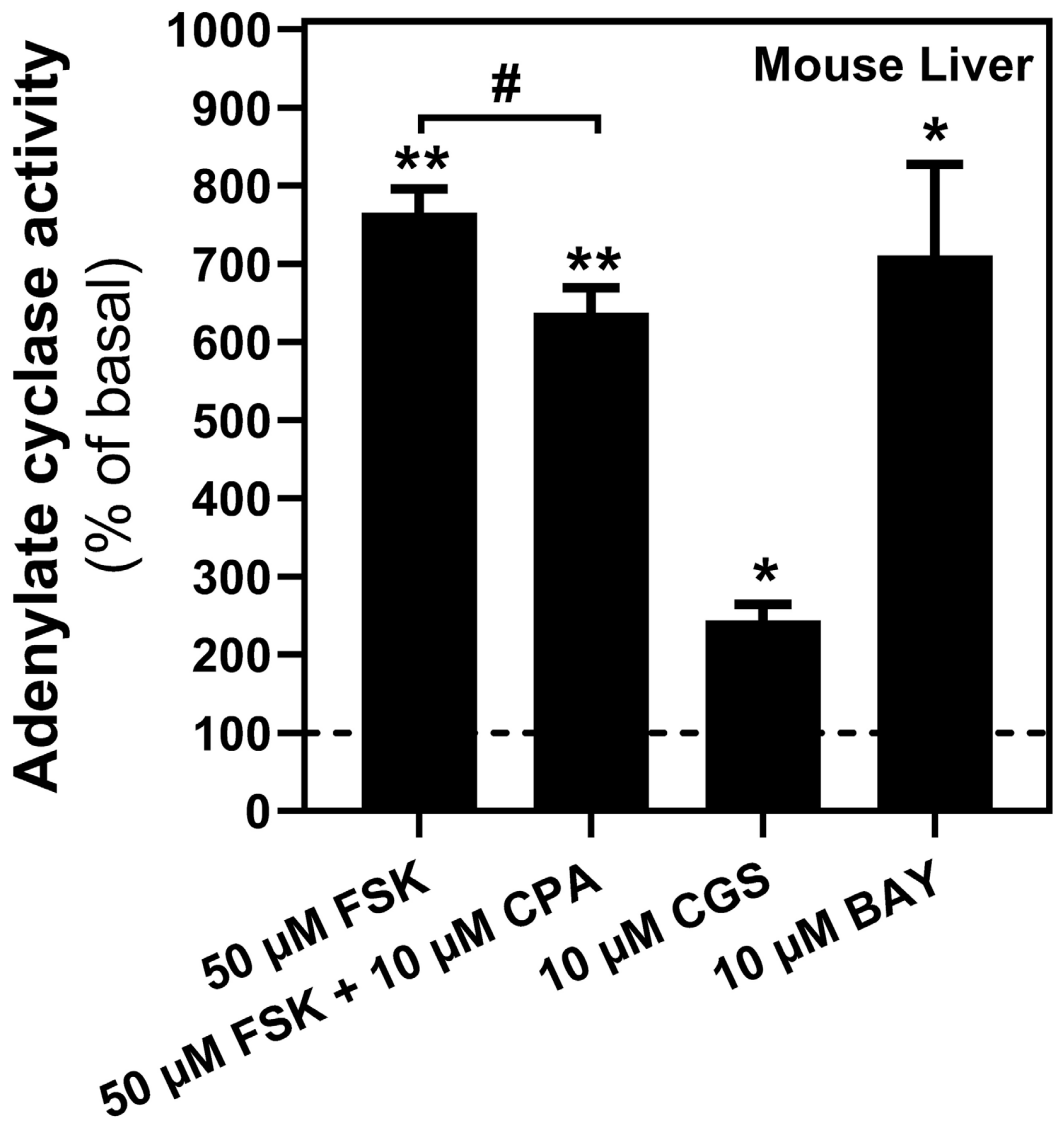
** Adenylyl cyclase activity in mouse liver mitochondria.** Mitochondria fraction was subjected to adenylate cyclase (AC) activity assays in the presence of indicated concentrations of adenosine A_1_ (CPA), A_2A_ (CGS 21680) or A_2B_ (BAY 60-6583) receptors' agonist. Adenosine A_1_-mediated AC inhibition was achieved in the presence of forskolin (FSK). * p < 0.05 and ** p < 0.01 significatively different from basal value (0.045 ± 0.02 pmol/mg prot·min). # p < 0.05 significatively different from FSK-stimulated activity according to the Student t-test. Data are the mean ± SEM of three independent assays performed with three mice.

Once the presence of adenosine receptors in the mitochondria from several tissues was confirmed, mainly on the outer membrane, we assessed the possible modulation of cAMP levels in the PM fraction from mouse liver through their coupling to G-proteins. As shown in Figure 5, forskolin induced a substantial cAMP accumulation, suggesting that the adenylyl cyclase/cAMP system was present and functional in this mitochondrial fraction. Furthermore, pharmacological stimulation of A_2A_ as well as A_2B_ receptors, particularly the latter, induced a strong increase in cAMP levels, whereas A_1_ receptor stimulation reduced the forskolin-dependent cAMP accumulation.

### Adenosine formation in mitochondria

**Figure 6:**
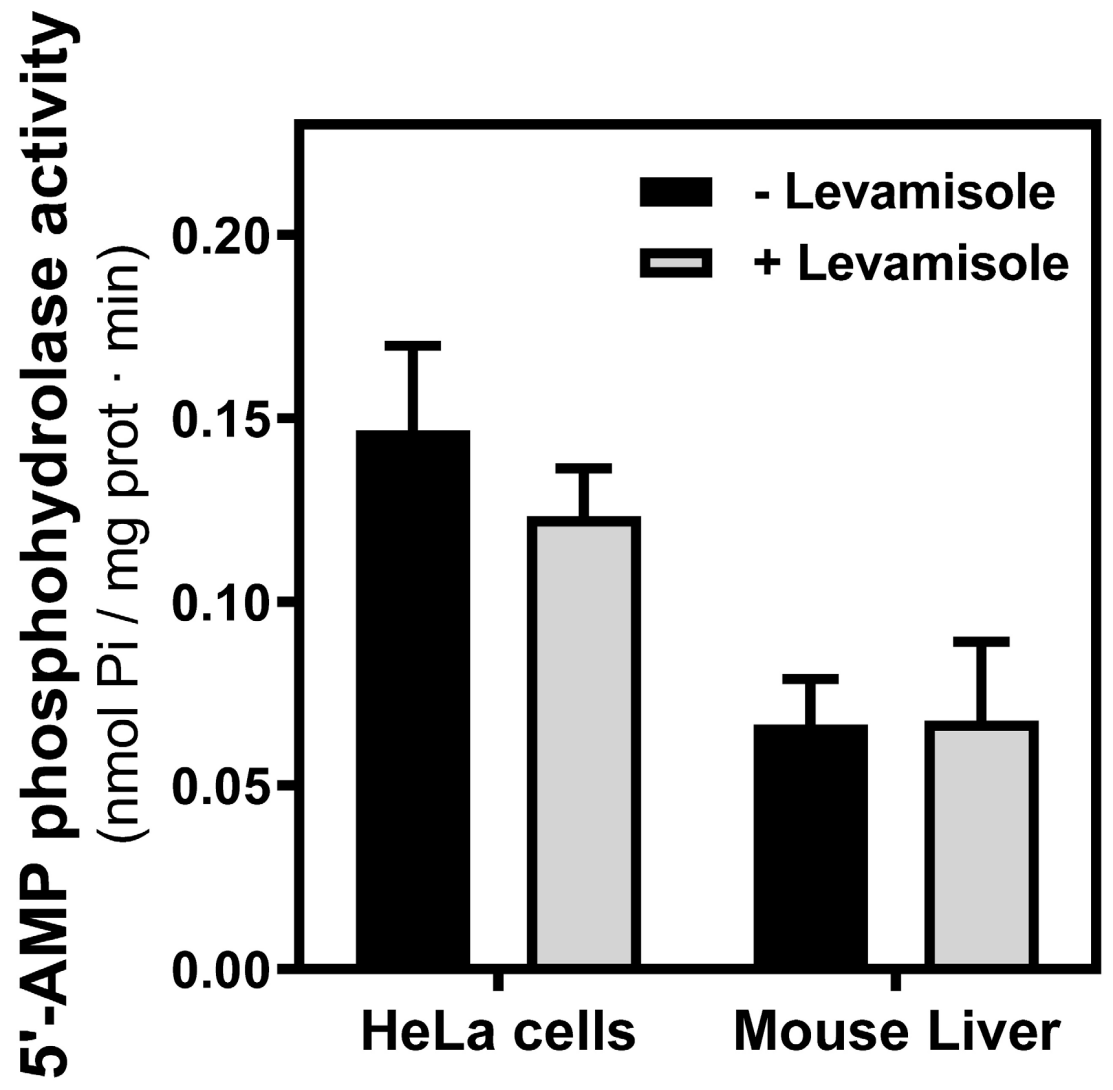
** 5'-AMP phosphohydrolase activity in mitochondria.** The hydrolysis of AMP was measured in mitochondria fraction from HeLa cells and mouse liver in the presence or the absence of levamisole, a phosphatase alkaline inhibitor. Data are the mean ± SEM of three independent assays.

Since CD73, a generating enzyme of adenosine, appears to be very near to adenosine receptors in the plasma membrane to facilitate adenosine receptor-mediated signaling 26, the 5′-AMP hydrolyzing activity was measured in PM fraction from both mouse liver and HeLa cells. These activities were insensitive to alkaline phosphatase inhibitor levamisole, indicating that the activity belonged to the CD73 enzyme (Fig. 6).

### Pharmacological stimulation of adenosine receptors modulates mitochondrial bioenergetics

**Figure 7:**
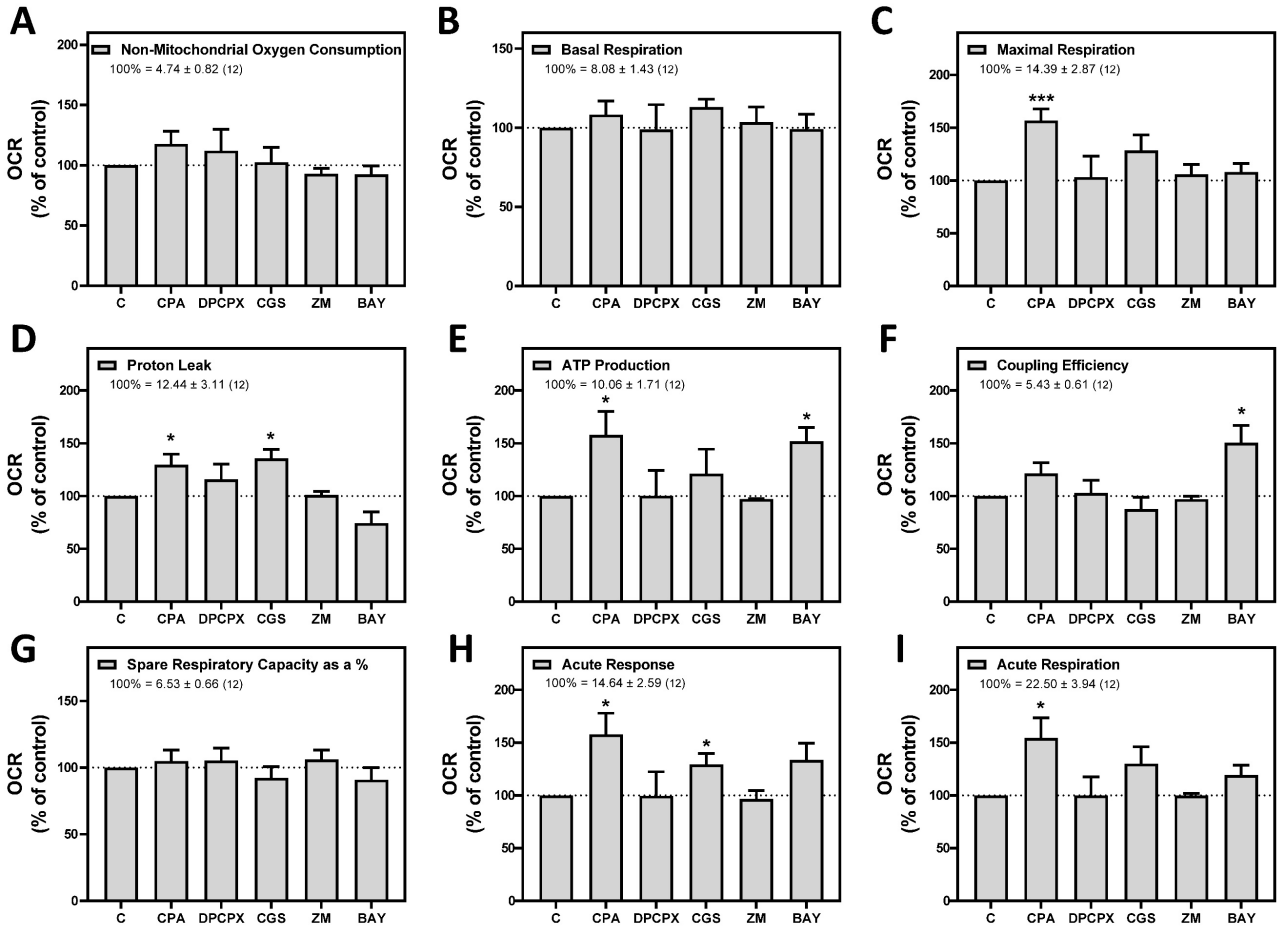
** Pharmacological stimulation of adenosine receptors modulates mitochondrial bioenergetics in HeLa cells**. Isolated mitochondria from HeLa cells were used to measure mitochondrial respiration by using SeaHorse analyzer XFp as described in “Materials and Methods”. Oxygen Consumption Rate (OCR) was determined in control cells (C) or in the presence of the A_1_ agonist CPA (CPA), the A_1_ antagonist DPCPX (DPCPX), the A_2A_ agonist CGS 21680 (CGS), the A_2A_ antagonist ZM 241385 (ZM) or the A_2B_ agonist BAY 60-6583 (BAY). (A) Non mitochondrial OCR. (B) Basal respiration. (C) Maximal respiration. (D) Proton leak. (E) ATP production. (F) Coupling efficiency. (G) Spare respiratory capacity. (H) Acute response. (**I**) Acute respiration. Each panel shows its corresponding OCR control value expressed as pmol/min·µg mitochondrial protein. Data are the mean ± SEM of twelve independent experiments. * p < 0.05 significantly different according to the Student t-test.

Next, we measured oxygen consumption rate (OCR) through Seahorse analysis to assess whether adenosine receptors can modulate mitochondrial energy metabolism. For this purpose, isolated crude mitochondria from HeLa cells were used to rule out the action of these receptors expressed on the cell surface. Injection protocol allowed the determination of several mitochondrial respiration-related parameters after incubation with selective A_1_, A_2A_, and A_2B_ agonists or antagonists (Scheme 2). Our data indicates that pharmacological manipulation of adenosine receptors did not alter non-mitochondrial OCR (Fig. 7A), basal respiration (Fig. 7B) or spare capacity (Fig. 7G). However, ATP production and coupling efficiency significantly increased in the presence of the selective agonist of A_2B_ receptor BAY 60-6583 (Fig. 7E and 7F, respectively), whereas proton leak (Fig. 7D) and acute response (Fig. 7H) were significantly higher after A_2A_ receptor activation with CGS 21680. Regarding the A_1_ receptor, several mitochondrial parameters such as maximal respiration (Fig. 7C), proton leak (Fig. 7D), ATP production (Fig. 7E), acute response (Fig. 7H), and acute respiration (Fig. 7I) were found significantly increased in the presence of CPA. On the other hand, the presence of the antagonists for A_1_ (DPCPX) or A_2A_ (ZM 241385) did not modulate any mitochondrial OCR parameter. These data might suggest that adenosine receptors may play a direct role in the modulation of mitochondrial energy metabolism.

### Mitochondrial morphology is modulated after exposure to adenosine receptor agonists

**Figure 8:**
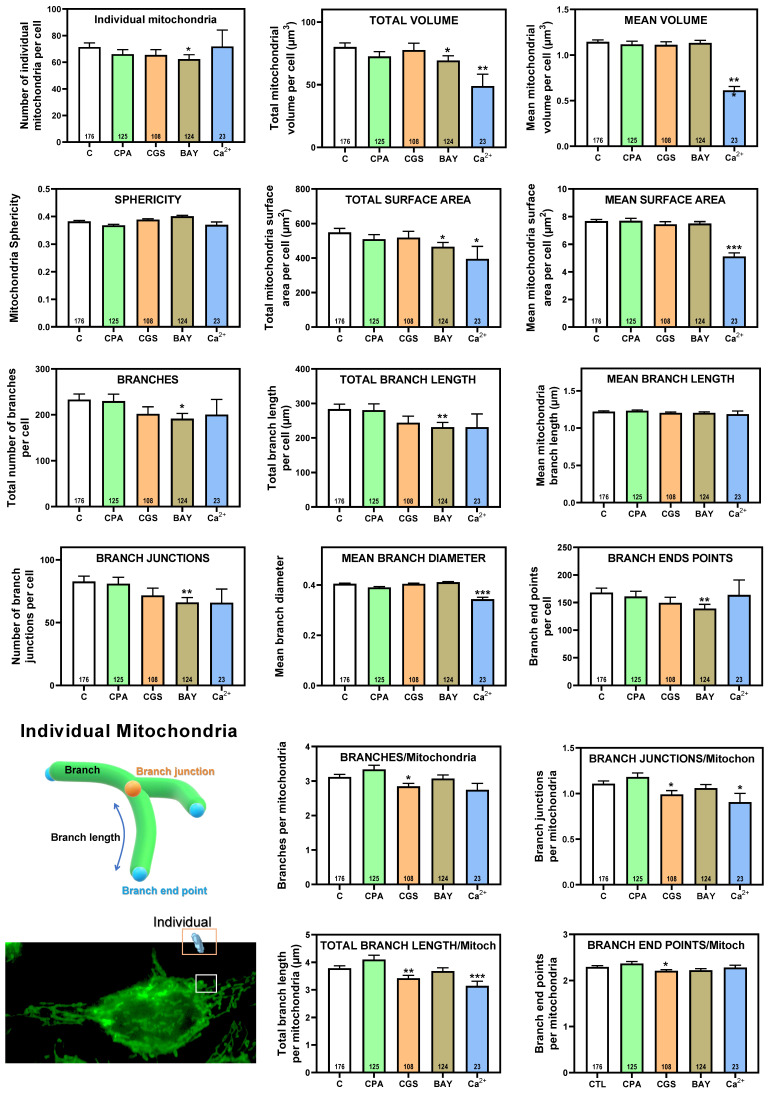
** 3D morphological analysis of individual mitochondria from HeLa cells.** HeLa cells were exposed for 24 h to 10 µM CPA, 10 µM CGS 21680, 10 µM BAY 60-6583, and 10 µM Ca^2+^ and the corresponding morphological parameters of the individual mitochondria were compared to control cells. Data are means ± SEM of n values (indicated within each graph bar). * p < 0.05, ** p < 0.01 and *** p < 0.001 significantly different according to the Student t-test.

**Figure 9:**
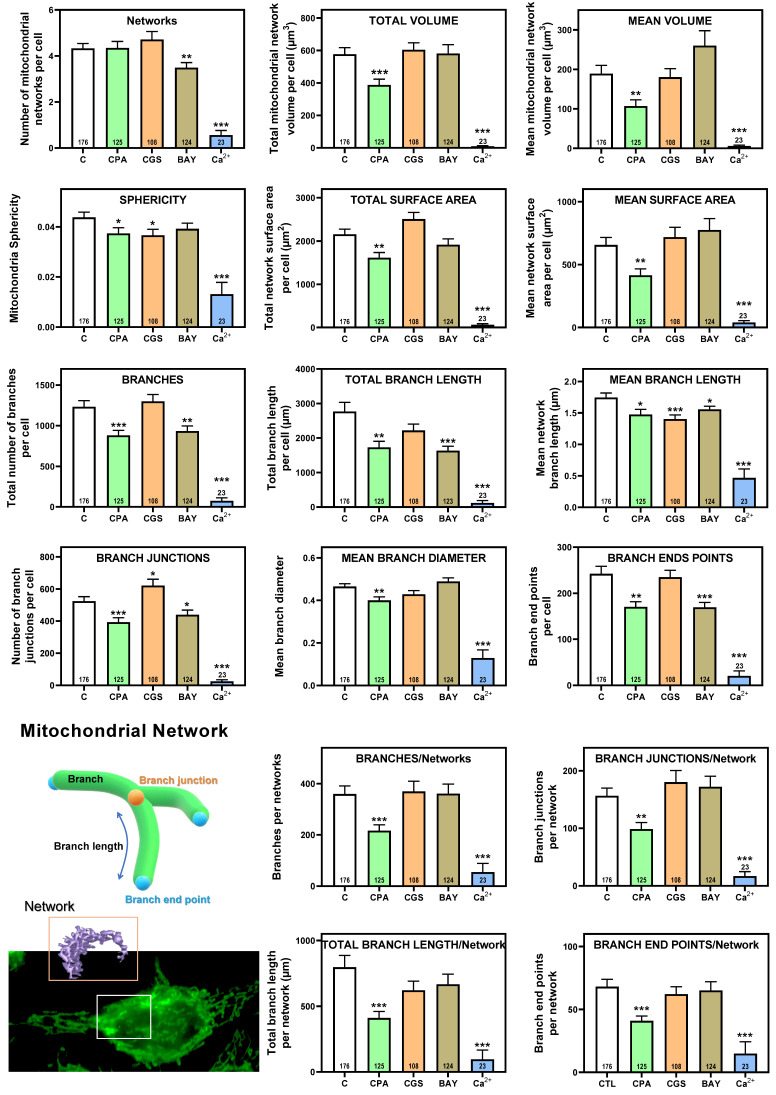
** 3D morphological analysis of mitochondrial network from HeLa cells.** HeLa cells were exposed for 24 h to 10 µM CPA, 10 µM CGS 21680, 10 µM BAY 60-6583, and 10 µM Ca^2+^ and the corresponding morphological parameters of the mitochondrial network compared to control cells. Data are means ± SEM of n values (indicated within each graph bar). * p < 0.05, ** p < 0.01 and *** p < 0.001 significantly different according to the Student t-test.

**Figure 10:**
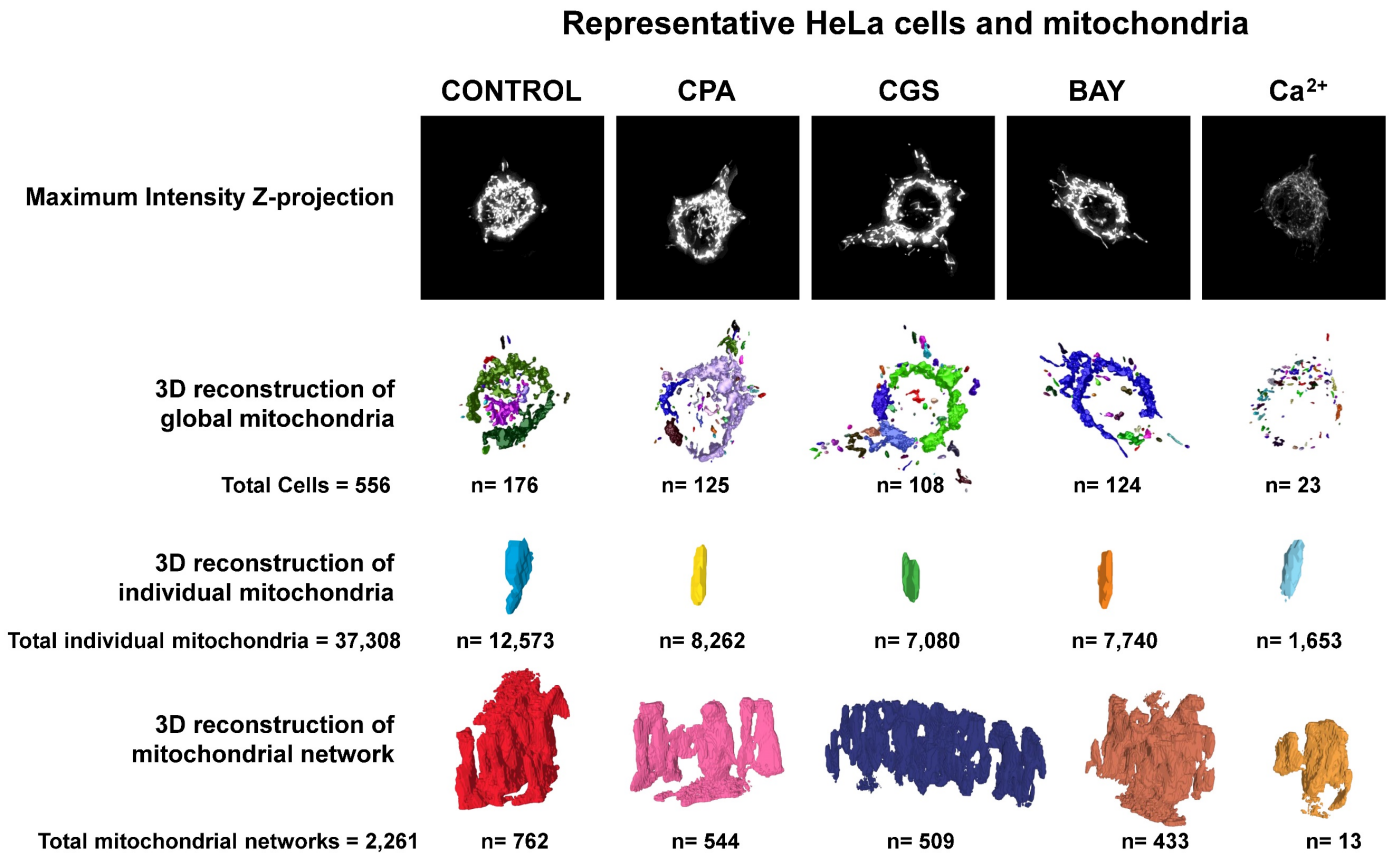
** Representative HeLa cells and mitochondria of the morphological parameters detected in control or agonist-exposed cells.** A total of 556 cells from 6 independent culture passages were analyzed, from which 37,3083 individual mitochondria and 2,261 mitochondrial networks were identified and analyzed. Maximum intensity Z-projections and 3D reconstructions correspond to the cells and mitochondria with morphological parameters close to medium values represented in Figures 8 (individual mitochondria) and 9 (mitochondrial networks), and Supplemental Figure 1 (global mitochondria).

Having shown that stimulation of adenosine receptors modulated mitochondrial energy metabolism in isolated mitochondria from HeLa cells, we examined mitochondrial morphology after a 24 hour exposure to selective adenosine receptor agonists. To this aim, live control or adenosine receptor-stimulated HeLa cells were incubated with the Mitotracker Green FM probe. Using time-lapse recording, dynamic changes in mitochondrial morphology were observed in control cells over a 4-minute growth period (Video S1). After 24 h treatment, z-stack images were captured from different control and treated cells and the corresponding 3D image analysis was performed (Scheme 3). This analysis revealed a significant diversity in mitochondrial size and shape. For clarity, we then decided to only distinguish between individual mitochondria (morphology with branch junctions ≤ 1 and branches ≤ 2) and networks (morphology with greater complexity, branching and volume). Negligible morphological changes were observed in individual mitochondria (Figure 8) in cells treated with adenosine A_2_ receptors agonists, mainly the A_2B_ agonist BAY 60-6583 (Table 1). On the contrary, the morphology of the mitochondrial network (Figure 9) was remarkably altered after adenosine A_1_ receptor activation (CPA), and to a lesser extent after stimulation with A_2B_ agonist (BAY 60-6583) and A_2A_ agonist (CGS 210680) (Table 1). These morphological changes were similar to those observed when the 3D analysis was performed without differentiating between individual mitochondria and mitochondrial network (i.e. global mitochondria) as Figure S1 shows. Since Ca^2+^ overload has been reported to increase mitochondrial fission and fragmentation 27, cells were maintained at 10 µM Ca^2+^ for 24 h to promote mitochondrial network changes and thus serve as a positive control. In agreement, our results revealed that calcium exposure reduced the number of mitochondrial networks, with lower total volume and branching, among other modified parameters, than those networks analyzed in controls (Figures 8 and 9). Representative images of the different treated cells and their corresponding mitochondrial 3D reconstructions are shown in Figure 10.

## Discussion

Results exposed herein demonstrate the mitochondrial localization of adenosine A_1_ and A_2_ receptors in pure mitochondria fractions isolated from different sources (i.e. mouse brain and liver, human brain, and HeLa and SH-SY5Y cells), particularly in the outer mitochondrial membrane. Adenylyl cyclase activity assays revealed that these receptors seem to be fully functional and coupled to their canonical G-proteins in this organelle similar to those described on the cell surface. Moreover, exposure of isolated mitochondria to selective A_1_, A_2A_, and A_2B_ receptors agonists suggest that these receptors could act as potential modulators of mitochondrial energy metabolism since ATP production and coupling efficiency significantly increased in the presence of BAY 60-6583 (A_2B_) whereas proton leak and acute response were significantly higher with CGS 21680 (A_2A_). Also, proton leak, ATP production, acute response, and acute respiration were found significantly increased in the presence of CPA (A_1_). Interestingly, different morphological changes were also detected in the mitochondrial structure in HeLa cells exposed to these receptors' agonists, probably contributing to the modulation of energetic metabolism.

### Localization of GPCRs beyond the plasma membrane

The classical view of GPCRs as signaling transducers localized at the plasma membrane is changing. In addition to the cell surface, many GPCRs localize and signal through both non-G protein and G protein effectors in compartments along the biosynthetic pathway and the endolysosomal pathway 28, 29. These intracellular membranes include endosomes, nucleus, Golgi and endoplasmic reticulum apparatuses, mitochondria, and cell division compartments (centrosomes, spindle midzone, and midbodies) 29. Therefore, processes such as intracellular trafficking, internalization, and recycling of GPCRs may contribute to a more complex signaling role in addition to the regulation of receptor desensitization and expression at the plasma membrane. In agreement, it has been proposed a new model of GPCR signaling to consider the ability of receptors to change their signaling properties depending on their subcellular localization 6. Different new terms such as “location bias”, “spatial bias,” or “spatial encoding” raised in recent years to refer to the role of receptor compartmentalization in their signaling functionality beyond the plasma membrane and their implication in numerous physiological and pathophysiological processes 30, 31. Moreover, intracellular GPCR signaling may contribute to improving therapeutic strategies against several diseases 32. Thus, targeting intracellular GPCRs would be a promising strategy for the treatment of pain 33 or noncommunicable diseases such as heart disease, cancer, chronic respiratory disease, and diabetes 34.

### Mitochondrial GPCRs

Accumulating evidence strongly suggests the mitochondrial localization of different GPCRs, particularly in the outer membrane of mitochondria, in a large variety of tissues and cells that display distinct biological functions. One of the most explored mitochondrial receptors is CB1. Marsicano's group first reported that mitochondrial CB1 receptor activation in the brain reduced cAMP/PKA signaling, thus lowering the respiratory-chain complex I activity and modulating the neuronal energy metabolism 7, which was then associated with a detrimental effect on cognitive functions 35. Moreover, astroglial CB1 receptors associated with mitochondrial membranes altered glucose metabolism and lactate production, leading to impaired neuronal functions and social behavior 36. Furthermore, both angiotensin II receptor type 1 (AT1) and 2 (AT2) were found in the mitochondria of dopaminergic neurons regulating superoxide production and mitochondrial respiration. In this work, the authors described a modulation of mitochondrial angiotensin receptors associated with aging, which might be involved in mitochondrial dysfunction 10. Other mitochondrial GPCRs are purinoceptor 1 like receptor (P2Y1), Purinoceptor 2 like receptor (P2Y2), 5-hydroxytrptamine receptor (5-HTR3 and 5-HTR4), and melatonin MT1 receptor (MT1R) 29. Whether or not GPCRs are constitutively expressed in mitochondria remains unclear. A trafficking pathway between the plasma membrane and mitochondria via clathrin-mediated endocytosis has been reported in astrocytes for styryl pyridinium FM dyes without early endosome (EE) participation but directly trafficked to mitochondria via microtubule-dependent active transport 37. The targeting mechanism of endocytosed receptors to mitochondria could also be EE-dependent and characterized by the transient “kiss and run” interactions between endosomes and mitochondria, as it has been reported for human salivary histatin 1 in human buccal epithelial carcinoma cells line HO1N1 38. In line with this, it has been reported that GPR35 (an orphan GPCR) is trafficked from the plasma membrane to the outer mitochondria membrane when bound by kynurenic acid or the GPR35 agonist pamoic acid 11. Consistently, mitochondrial localization of the ionotropic P2X7 receptor increased upon agonist exposure 39. Furthermore, very recently it has been reported the A_2B_ translocation over time from liver plasma membrane to mitochondria after acetaminophen overdose injection to C57BL mice 40. Interestingly, delayed treatment with BAY 60-6583 enhanced the mitochondrial localization of A_2B_, indicating that receptor activation modulates A_2B_ trafficking.

Another possibility is that a yet unknown signal targets GPCR directly to the mitochondria. In line with this, Fasciani et al. 41 recently identified an internal ribosome entry site (IRES) element within the third intracellular loop of the human M2R that directs the expression of a C-terminal M2R fragment (containing the C-terminal portion of the i3 loop, transmembrane domains 6 and 7, and the cytoplasmic C-terminal receptor sequence) to the mitochondria.

### Presence of adenosine receptors in mitochondria

The diversity of signaling mechanisms in the ever-growing list of active intracellular GPCRs comprises the coupling to Gαs, Gαq, and Gαi proteins 30. Moreover, the existence of the AC/cAMP/PKA signaling cascade inside the mitochondria has been corroborated by numerous studies as reviewed elsewhere 42. This is especially interesting since many GPCRs, including adenosine receptors, act through this transduction pathway. In this sense, it has been described that increased cAMP levels by soluble adenylyl cyclase (sAC) activates PKA, which in turn, phosphorylates complex I of the respiratory chain, leading to a higher mitochondrial respiration 17. Furthermore, β-arrestin, another key participant in GPCR signaling, has been also found within mitochondria, particularly in the intermembrane space, in mouse brain 9 and rat cardiomyocytes 43, suggesting that GPCR signaling system is fully available and functional in this organelle.

There are very few previous studies that have suggested the presence of adenosine receptors in the mitochondria. Thus, the adenosine A_1_ receptor was found in C57BL/6 mouse brain mitochondria 44 and A_2B_ was found in the mitochondria, likely in the outer membrane, from rat cardiomyocytes 43. In the present work, we have demonstrated the mitochondrial localization of adenosine A_1_, A_2A_, and A_2B_ receptors in pure mitochondria fractions isolated from different sources.

**Scheme 4:**
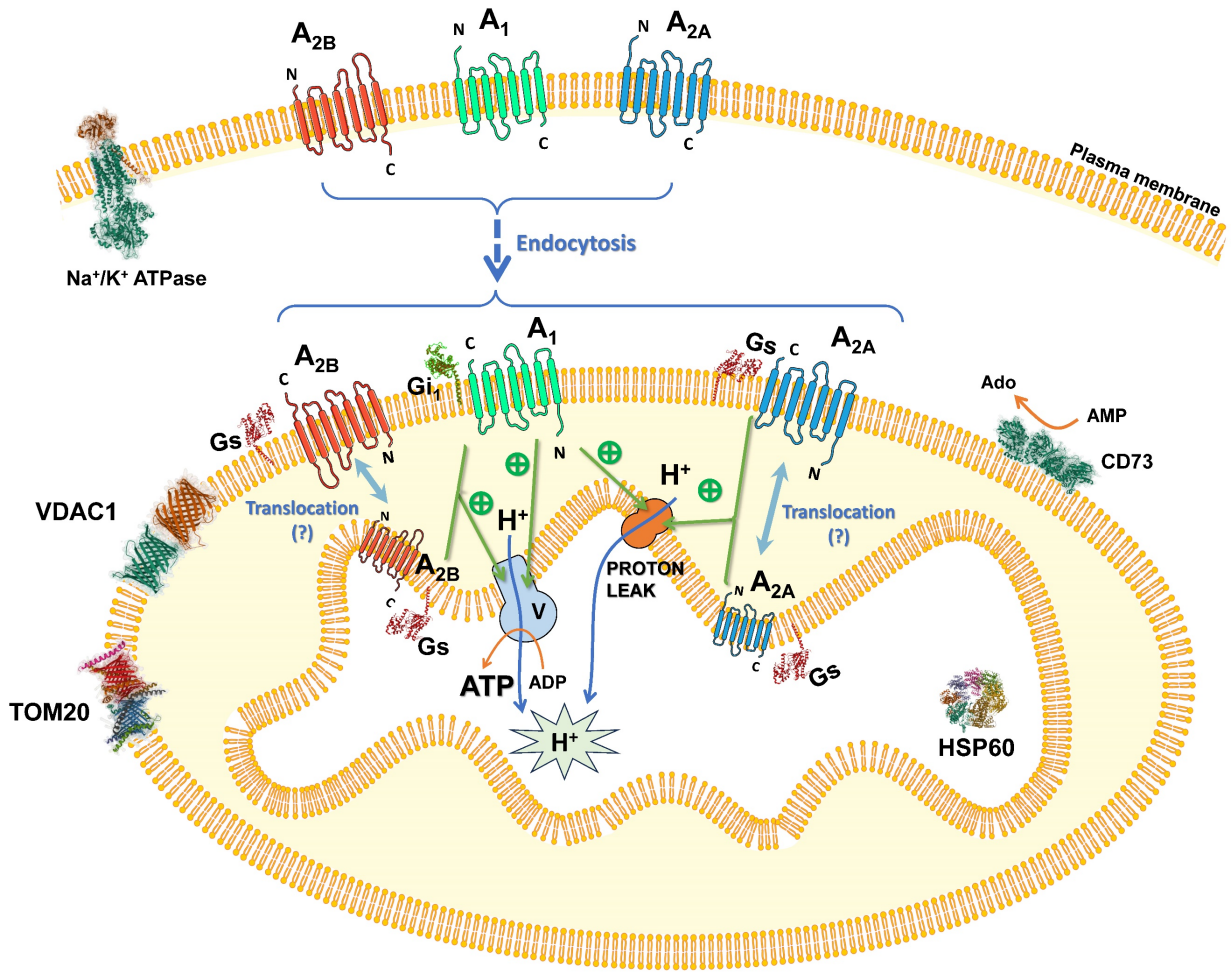
** Proposed organization and role of adenosine receptors in mitochondria.** Adenosine receptors are localized in the plasma membrane and may undergo internalization via endocytosis, targeting the mitochondria and localizing mainly to the outer mitochondrial membrane, with the possibility of being translocated to the inner mitochondrial membrane. Within this context, they can be activated by mitochondrially derived adenosine, thereby modulating ATP synthesis through an as-yet-uncharacterized mechanism. This regulatory pathway may involve modifications in mitochondrial morphology and/or the function of specific electron transport chain complexes. Consequently, the adenosine receptors' signaling efficacy is likely augmented by this emerging bioenergetic function.

Regarding ectonucleotidases in mitochondria, Raatikainen and colleagues identified in 1992 the presence of CD73 activity in rat liver mitochondria wherein adenosine production was detected 45. We also detected a weak activity of CD73 in pure mitochondria fraction from both HeLa cells and mouse liver compared to the enzymatic activity detected in the plasma membrane 46. CD73 appears to be very near to adenosine receptors in the plasma membrane to facilitate adenosine receptor-mediated signaling 26. A similar organization could take place in mitochondria. Furthermore, it has been reported the existence of an equilibrative adenosine transport system in mitochondria from rat testis 47 and the mitochondrial expression of the human equilibrative nucleoside transporter 1 (hENT1) in human livers 48, which might mediate the passage of adenosine formed in the mitochondria to the cytoplasm. All these observations might suggest a mitochondrial localization of an enzymatic system involved in the regulation of ATP-derived adenosine likely to modulate mitochondrial functions through adenosine receptors signaling located on this organelle. This idea may be supported by a previous work that also identified the enzymatic machinery to produce melatonin within mouse brain mitochondria. Yet, an “automitocrine” signaling was hypothesized since mitochondria-derived melatonin activated mitochondrial MT1 receptors, thus allowing it to contribute to the neuroprotective action of this neurotransmitter by reducing the cytochrome C release 9. Interestingly, mitochondrial GPCRs have been mainly localized in the outer membrane 9, 49-51. MT1 receptors have been proposed to be oriented with their signaling part toward the intermembrane space 9. However, the precise topology of GPCRs within the OMM is unclear yet. A hypothetical organization of adenosine receptors in the mitochondria is depicted in Scheme 4.

### Mitochondrial adenosine receptors are functional

Our results confirm that adenylyl cyclase activity is responsive to adenosine A_1_, A_2A_, and A_2B_ receptor activation in isolated mitochondria from mouse liver. Despite the growing evidence linking adenosine receptors to metabolic disorders 52, 53, the precise mechanisms remain unclear. As stated above, the adenosine A_1_ receptor was found in mouse brain mitochondria and its genetic depletion led to reduced mitochondrial activity 44, which might be consistent with our data showing an increase in several mitochondrial parameters (maximal respiration, proton leak, and ATP production among others) after CPA incubation. The functionality of adenosine A_2B_ receptors found in mitochondrial membranes from rat cardiomyocytes 43 was not explored. However, it has been recently demonstrated that A_2B_ activation increases energy expenditure and oxygen consumption and counteracts obesity in mice. Yet, genetic depletion of A_2B_ reduced body temperature and oxygen consumption 54. This might explain our findings showing, for the first time, that mitochondrial A_2B_ activation induced a higher rate of ATP production and mitochondrial coupling efficiency. Concerning adenosine A_2A_ receptors, they have not been described within mitochondria until now, however, pharmacological stimulation enhanced the mitochondrial metabolism of primary mouse chondrocytes, which agreed with reduced oxidative phosphorylation and altered mitochondrial ultrastructure in mice lacking A_2A_ 55. Moreover, the A_2A_ selective agonist, CGS 21680, increased O_2_ consumption and thermogenesis in mouse adipose tissue. These observations were significantly reduced in A_2A_-KO mice showing a reduced body temperature and lower O_2_ consumption 56. In contrast, our results showed that CGS 21680 increased mitochondrial acute response and proton leak. Proton leak (the migration of protons to the mitochondrial matrix independent of ATP synthase) has been associated with: i) mitochondrial damage; ii) thermogenesis linked to uncoupling proteins (UCPs) 57; and iii) a lower reactive oxygen species 7 production to minimize oxidative damage 58.

### Mitochondrial adenosine receptors modulate mitochondrial respiration and morphology

Mitochondria is a highly dynamic organelle, with constant shape changes by fission and fusion processes as key mediators of cellular function 59. In agreement, imbalanced mitochondrial dynamics are observed in a range of diseases including neurodegenerative and cardiac disorders 16. Since mitochondrial morphology is continuously changing within a cell, its quantification is a challenge given the heterogeneity in mitochondrial shape, with different lengths and degrees of branching. Consequently, the number of terms employed to identify and classify various mitochondrial forms is vast, and these terms are not fully standardized, making the results highly subjective and difficult to translate between laboratories 60. To perform this morphological analysis, numerous tools and applications have been developed, as reviewed in 61. Among these, we adopted the analysis procedure illustrated in Scheme 3, including a critical step concerning the segmentation of fluorescence images with a machine learning based 3D segmentation of mitochondria 23. This analysis pipeline allows us to approximate the global state of the mitochondrial network in a cell subjected to a specific treatment. In the present work, to avoid confusion in the terminology of the detected mitochondrial forms, we decided to consider only two types: individual forms (small, non-branching) and mitochondrial networks (larger volume, with branching).

Our results suggest that in HeLa cells A_1_ receptor activation with CPA induces a greater number of changes in both respiratory capacity and mitochondrial morphology compared to A_2B_ receptor activation with BAY 60-6583. On the other hand, despite A_2A_ receptor activation with CGS 21680 enhances proton leak and acute response, no apparent changes in mitochondrial morphology were observed. The potential correlation between morphological changes and mitochondrial respiratory capacity is difficulted by the distinct nature of the samples tested (isolated mitochondria versus whole live cells) and the exposure times to receptors agonists employed in OCR measurement (75 min) and morphological analysis (24 hours of treatment).

Energetically demanding cellular activities and extreme or pathological conditions may change mitochondrial fission and fusion rates 62. Thus, an increased proportion of inter mitochondrial junctions and density of the internal mitochondrial membrane may upregulate ATP production 23 as well as the remodeling of mitochondrial cristae and its impact on the embedded electron transport system 63. Alterations in mitochondrial morphology were reported to alter the proton conductance as well 64 and could be responsible for the increased proton leak detected after A_1_ receptor activation. However, A_2A_ activation also increases proton leak but no apparent changes in mitochondrial morphology are detected. In HeLa cells, which natively express more A_2B_ than A_2A_ receptors, it has been reported that A_2B_ receptors physically interact at the plasma membrane with A_2A_ forming A_2A_-A_2B_ heteromers where A_2A_ signaling appears to be completely blocked 65. Although GPCR oligomerization in mitochondria has not been described yet, it is tempting to speculate that our data could suggest that adenosine acting through both mitochondrial A_2A_ and A_2B_ receptors (free or heterodimerized) might modulate mitochondrial ATP production. The precise molecular mechanism by which adenosine receptor activation affects mitochondrial functionality and morphology will require substantial further investigation and is not addressed in depth in this article. However, it is intriguing to consider that adenosine receptors could play a significant role in potential therapeutic strategies aimed at restoring mitochondrial function, as is being considered for diseases such as cancer and Alzheimer's disease 62.

## Conclusion

Taken altogether, mitochondrial localization of adenosine receptors and their actions on this organelle (i.e. morphology changes and energy production) should be further considered as new targets for regulating mitochondrial dysfunction linked to aging, neurodegeneration, cancer, metabolic and inflammatory disorders, among others.

## Supplementary Material

Supplementary figure and video legend.

Supplementary video.

## Figures and Tables

**Table 1:** ** Summary of mitochondrial morphological changes detected in HeLa cells.** Increased (↑) or decreased (↓) values are indicated for a change of 10 to 30 percent (one arrow), a change of 30 to 50 percent (two arrows), or a change greater than 50 percent of the control value (three arrows). ≈, change less than 10 percent of the control value.

	Individual mitochondria morphology				Mitochondrial network morphology			
Parameter	CPA (A_1_)	CGS (A_2A_)	BAY (A_2B_)	Ca^2+^	CPA (A_1_)	CGS (A_2A_)	BAY (A_2B_)	Ca^2+^
Number	≈	≈	↓	≈	≈	≈	↓	↓↓↓
Total Volume	≈	≈	↓	↓↓	↓↓	≈	≈	↓↓↓
Mean Volume	≈	≈	≈	↓↓	↓↓	≈	↑↑	↓↓↓
Total Surface Area	≈	≈	↓	↓	↓	↑	↓	↓↓↓
Mean Surface Area	≈	≈	≈	↓↓	↓↓	≈	↑	↓↓↓
Branches	≈	↓	↓	↓	↓	≈	↓	↓↓↓
Total Branch Length	≈	↓	↓	↓	↓↓	↓	↓↓	↓↓↓
Mean Branch Length	≈	≈	≈	≈	↓	↓	↓	↓↓↓
Mean Branch Diameter	≈	≈	≈	↓	↓	≈	≈	↓↓↓
Branches / Mitochondria or Network	≈	≈	≈	↓	↓↓	≈	≈	↓↓↓
