# Supplementary material for: Mitochondrial Localization and Function of Adenosine Receptors
Source: Int J Biol Sci. 2025 Feb 10;21(5):1874–93. doi: 10.7150/ijbs.101930 (PMC11900819; doi:10.7150/ijbs.101930)
Supplement: Supplementary file 1 — Supplementary figure and video legend. [file ijbsv21p1874s1.pdf]

Figure S1. 3D morphological analysis of global mitochondria from HeLa cells. HeLa cells were exposed for 24 h to 10 μM CPA, 10 μM CGS 21680, 10 μM BAY 60-6583, and 10 μM Ca^{2+} and the corresponding morphological parameters of the global mitochondria (individual mitochondria and mitochondrial network) compared to control cells. Data are means ± SEM of n values (indicated within each graph bar). * $p < 0.05$, ** $p < 0.01$ and *** $p < 0.001$ significantly different according to the Student t-test.

Video S1. Mitochondria staining in HeLa cells. Control HeLa cells were exposed to Mitotracker Green FM to stain mitochondria and time-lapse was recorded with a 63x immersion oil objective for 4 minutes. Cells were grown in a microchamber (PeCon) to maintain a humidified atmosphere with 5% CO_2 at 37°C. The scale bar and relative time are included in the video.

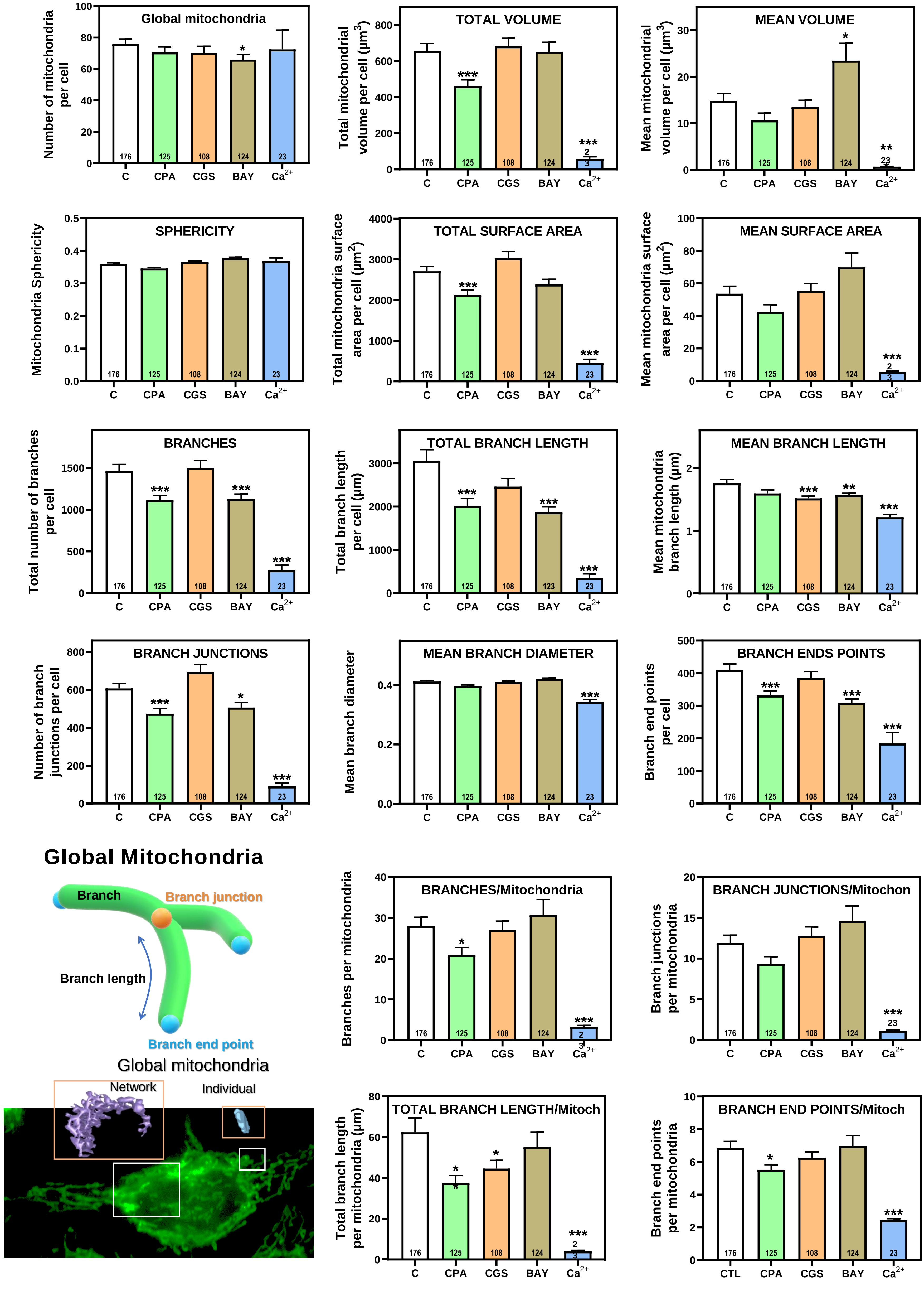
Global mitochondria
Number of mitochondria per cell
TOTAL VOLUME
Total mitochondrial volume per cell (µm3)
MEAN VOLUME
Mean mitochondrial volume per cell (µm3)
SPHERICITY
Mitochondria Sphericity
TOTAL SURFACE AREA
Total mitochondria surface area per cell (µm2)
MEAN SURFACE AREA
Mean mitochondria surface area per cell (µm2)
BRANCHES
Total number of branches per cell
TOTAL BRANCH LENGTH
Total branch length per cell (µm)
MEAN BRANCH LENGTH
Mean mitochondria branch length (µm)
BRANCH JUNCTIONS
Number of branch junctions per cell
MEAN BRANCH DIAMETER
Mean branch diameter
BRANCH ENDS POINTS
Branch end points per cell
Global Mitochondria
Branch
Branch junction
Branch length
Branch end point
Global mitochondria
Network
Individual
BRANCHES/Mitochondria
Branches per mitochondria
BRANCH JUNCTIONS/Mitochon
Branch junctions per mitochondria
TOTAL BRANCH LENGTH/Mitoch
Total branch length per mitochondria (µm)
BRANCH END POINTS/Mitoch
Branch end points per mitochondria
C
CTL
CPA
CGS
BAY
Ca2+
176
125
108
124
123
23
